# Microbial community succession during tobacco fermentation reveals a flavor-improving mechanism

**DOI:** 10.3389/fbioe.2025.1627842

**Published:** 2025-07-21

**Authors:** Jingjing Zhu, Jitao Wang, Zichao An, Chen Shen, Hongxu Dong, Haojun Wang, Zhenxing Peng, Bo Yang, Juanjuan Liu, Xiaojie Wang, Zemin Fang

**Affiliations:** ^1^ School of Life Sciences, Anhui University, Hefei, Anhui, China; ^2^ Anhui Key Laboratory of Biocatalysis and Modern Biomanufacturing, Hefei, Anhui, China; ^3^ China Tobacco Anhui Industrial Co. Ltd., Hefei, Anhui, China

**Keywords:** flue-cured tobacco, microbial fermentation, tobacco quality, microbial community analysis, metabolites

## Abstract

**Introduction:**

Flue-cured tobacco (FCT) requires fermentation to increase quality, with microorganisms playing a key role. However, microbial succession and functions during long-term fermentation remain unclear. Artificial microbial fermentation, which is more controllable and efficient, focuses on mining functional strains to optimize the process.

**Methods:**

In this study, the microbial community structure and function of FCT fermentated for 0–4 years were analyzed, and the changes of metabolites in tobacco leaves in different years were analyzed. Functional microorganisms were screened, and their potential for application in FCT fermentation was evaluated.

**Results:**

The results revealed that the FCT aging process was typified by the metabolic, transformative, and synthetic processes of alkaloids, their derivatives, and benzene ring compounds. Microbial succession leads to changes in metabolites, with *Escherichia*, *Bacillus*, *Enterococcus*, *Alternaria*, *Vibrio*, and *Halomonas* playing crucial roles in the breakdown of fundamental substances during the initial year of FCT fermentation. *Bacillus*, one of the dominant and highly active genus of the microbial community on the surface of tobacco leaves, exhibits significantly increased abundance during FCT fermentation and improves the aroma and flavor of tobacco leaves by participating in aromatic amino acid metabolism. After 15 days of treatment with a combination of four *Bacillus* strains (*Bacillus altitudinis* YS193, *Bacillus pumilus* YH186, *Bacillus tequilensis* YS154, and *Bacillus velezensis* YS157), the sugar‒nicotine ratio of FCT was effectively optimized, the sensory flavor was enhanced, and the levels of volatile compounds associated with the aromatic amino acid metabolic pathway were significantly increased.

**Discussion:**

This study reveals the critical role of microbial succession in FCT fermentation and demonstrates that targeted inoculation of functional *Bacillus* strains can significantly improve tobacco quality by modulating key metabolic pathways, providing a scientific basis for artificial microbial fermentation in tobacco processing.

## 1 Introduction

Tobacco is a worldwide economic crop that is typically manufactured into flue-cured tobacco (FCT) and ultimately into cigarettes (Wu et al., 2021b). After harvesting and roasting, FCT needs to undergo fermentation, which is referred to as aging and necessitates fermentation at particular temperature and humidity levels for a prolonged duration to increase its quality (Wang et al., 2019; Wu et al., 2021a) and diminish unpleasant odors before its utilization in cigarette products (Huang et al., 2010). FCT is usually fermented for approximately two to 3 years under natural conditions (20°C–30°C, 65%–75% relative humidity) to mature the product (Huang et al., 2010), which is too slow for some types of FCT to meet the desired product standards promptly and will undoubtedly lead to contamination during fermentation, ultimately increasing the cost of raw material preparation. There is a growing emphasis on developing more effective approaches to increase FCT fermentation efficiency to overcome the limitations of present solutions (Peng et al., 2016a; Zhang et al., 2024b).

FCTs are prepared mainly by a spontaneous fermentation process carried out by complex communities of microorganisms. Usually, metabolic processes performed by microbes, enzymes, and chemical oxidation are proposed to break down macromolecules, including proteins and starch, during fermentation to generate small molecules or compounds with aromatic flavors, with microbes playing critical roles in improving FCT quality (Chattopadhyay et al., 2023). Because the succession of microbial communities in FCT is dynamic during fermentation, it is particularly important to study the relationships between microorganisms and metabolites and to identify the core microorganisms that have positive effects on the flavor components of FCT.

In recent years, although there have been several studies on the microbial community involved in tobacco fermentation, these investigations were performed using short-term aged samples and conducted under laboratory conditions, failing to replicate the actual microbial changes that occur during real tobacco fermentation in industrial production (Wu et al., 2023c; Zhang et al., 2023). The actual succession process of the microbial community and metabolites over multiple years of production remains unclear. Thus, more investigations should be performed to determine the types of microorganisms and metabolites that play roles in the FCT fermentation process to improve the control of the industrial fermentation production process and the quality of the end product (Qin, 2014; Li et al., 2020a; Wu et al., 2022d).

Some microorganisms have been isolated and identified to gain a deeper understanding of their role in improving the quality of aged tobacco leaves. For example, the starch-degrading *Bacillus subtilis* strain BS3 was successfully isolated from tobacco leaf surfaces and rhizosphere soil. It regulates the composition of dominant bacteria by regulating the composition and diversity of the leaf microbial community, promotes the production of starch and cellulose-related enzymes, and significantly accelerates starch degradation in tobacco leaves (Zhang et al., 2024c). Moreover, a *Bacillus amylolitica* W6-2 strain with pectin degradation ability was screened from the surface of Yunnan flue-cured tobacco leaves. Pectinase produced by W6-2 destroyed macromolecular material connections in the cytoskeletal structure of the tobacco leaf cell wall, effectively decomposing pectin to form small-molecule reducing sugars, increasing the content of reducing sugars, reducing the irritability of FCT and improving the sweetness of FCT (Weng et al., 2024). The accumulation of reducing sugars contributes to the formation of Maillard reaction products that enhance aroma and flavor, thereby providing nutty, sweet, or popcorn flavors that help improve the softness and aroma intensity of baked FCT (Hinneh et al., 2018). *Bacillus amyloliquefaciens*, which has high α-amylase activity, and *Bacillus kochii*, which has high neutral protease activity, were selected from the tobacco leaf microbial community. During cofermentation with these two strains, the contents of most Maillard reaction products and terpenoid metabolites increase, which promotes the aroma and softness of FCT and reduces irritation (Wu et al., 2021b). It is evident that different bacterial fermentations have varying effects on FCT. However, past research has provided limited insights into the roles of various microorganisms during the FCT fermentation process. There remains a vast potential for discovering microorganisms and their combinations that could either accelerate the fermentation of FCT or directly enhance its quality.

In this study, the differentially abundant metabolites and microbial community composition in FCTs aged 0–4 years were analyzed. We screened four strains of *Bacillus* capable of accelerating and improving the quality of FCT on the basis of their amylase, protease, and cellulase activities. Our results suggest that in the process of tobacco fermentation, microbial succession leads to changes in tobacco metabolites. These findings may facilitate the development of microbial starters and improve tobacco fermentation quality.

## 2 Materials and methods

### 2.1 FCT samples

Five batches of FCT samples of the cultivar “Yunyan 97”, sourced from tobacco-growing regions in southern Anhui Province, were provided by China Tobacco Anhui Industry Co., Ltd. Samples were procured at five time points to evaluate the effects of aging time on FCT quality, including re-roasting (namely, RR) and aging for one--four years (A1--A4) (Supplementary Table S1). Each sample of approximately 500 g was randomly withdrawn via a five-point sampling strategy, with four from the corners and one from the center of each storage package. Each sample was mixed evenly, placed into a sterile bag for sealing, transported to the laboratory immediately and stored at −20°C for the following experiments.

### 2.2 Metabolite extraction and liquid chromatography‒tandem mass spectrometry (LC‒MS) analysis of FCT samples

Each FCT sample of 1.0 g was frozen in liquid nitrogen and ground into a fine powder with a mortar and pestle. Then, 10 mL of methanol/acetonitrile/H_2_O (2:2:1, v/v/v) was added to the homogenized solution for metabolite extraction. The mixture was centrifuged for 20 min (14,000 × g, 4°C), and the supernatant was dried in a vacuum centrifuge. After that, each sample was redissolved in 1.0 mL of acetonitrile/water (1:1, v/v) and centrifuged at 14,000 × g at 4°C for 15 min, and the resulting supernatant was used for LC‒MS analysis.

Analysis was performed via UHPLC (1,290 Infinity LC, Agilent Technologies) coupled to a quadrupole time-of-flight system (AB Sciex TripleTOF 6,600). Briefly, 2 μL of each sample was separated via a C-18 column at 40°C, with the flow rate set at 0.4 mL/min. Mobile phase A consisted of 25 mM ammonium acetate and 0.5% formic acid in water, and mobile phase B consisted of methanol. The gradient elution procedure was as follows: 0–0.5 min, 5% B; then, B was changed to 100% linearly from 0.5–10 min and maintained at 100% from 10–12 min. B was changed linearly from 100% to 5% from 12.0–12.1 min and maintained at 5% from 12.1–16 min. The ESI source conditions were set as follows: Ion Source Gas1 (Gas1) as 60, Ion Source Gas2 (Gas2) as 60, curtain gas (CUR) as 30, source temperature: 600°C, and ion spray voltage floating (ISVF) ± 5500 V. In MS-only acquisition, the instrument was set to acquire over the m/z range of 60–1,000 Da, and the accumulation time for the TOF‒MS scan was set at 0.20 s/spectra. For auto MS/MS acquisition, the instrument was set to acquire ions over the m/z range of 25–1,000 Da, and the accumulation time for the product ion scan was set at 0.05 s/spectra. The product ion scan was acquired via information-dependent acquisition (IDA) in high-sensitivity mode. The parameters were set as follows: the collision energy (CE) was fixed at 35V ± 15 eV; the declustering potential (DP) was 60 V (+) and −60 V (−); and the isotopes within 4 Da were excluded, and the number of candidate ions to monitor per cycle was 10.

The raw data were analyzed via ProteoWizard MSConvert and XCMS software (Sud et al., 2007; Shin et al., 2019). For peak picking, the following parameters were used: centWave m/z = 10 ppm, peak width = c (10, 60), and prefilter = c (10, 100). For peak grouping, bw = 5, mzwid = 0.025, and minfrac = 0.5. CAMERA (Collection of Algorithms of MEtabolite pRofile Annotation) was used to annotate isotopes and adducts. For the extracted ion features, only the variables with more than 50% nonzero measurement values in at least one group were retained. Metabolic compound identification was performed by comparing the accuracy of the m/z values (Luo et al., 2017; Gu et al., 2018).

Principal component analysis (PCA) and orthogonal partial least squares discriminant analysis (OPLS-DA) were conducted via the R package (Kolde R, 2025). Model robustness was assessed through 7-fold cross-validation and response permutation testing. The contribution of each variable to classification was determined by calculating the variable importance in the projection (VIP) value within the OPLS-DA model.

### 2.3 Metagenomic sequencing and analysis

Approximately 5 g of each FCT sample was placed into 500-mL shake flasks containing 100 mL of sterile phosphate-buffered saline buffer (PBS, pH 7.5). Microbes on the leaves were collected by shaking the flasks at 4°C for 1 h and centrifuging the eluent at 500 × g for 2 h. The FCT samples were rinsed repeatedly three times with PBS buffer. The total washing mixture was collected and enriched with 0.22 μm filter membranes to enrich the bacteria. The genomic DNA of microbes from 5 FCT samples was extracted via the FastDNA SPIN Kit (MP Biomedicals, Solon, OH, United States) following the manufacturer’s instructions and stored at −20°C prior to further assessment. The quantity and quality of the extracted DNA were measured via a NanoDrop ND-1000 spectrophotometer (Thermo Fisher Scientific, Waltham, MA, United States) and agarose gel electrophoresis, respectively. The extracted microbial DNA was processed to construct metagenome shotgun sequencing libraries with insert sizes of 400 bp via the Illumina TruSeq Nano DNA LT Library Preparation Kit, and sequencing was carried out on the Illumina HiSeq X-ten platform (Illumina, San Diego, CA, United States) with the PE150 strategy at Personal Biotechnology Co., Ltd. (Shanghai, China) following the manufacturer’s instructions.

Raw sequence reads that contained sequencing adapters (overlapping >15 bp) and low-quality reads (Q value <38) were processed to obtain quality-filtered reads for further analysis. Briefly, adapters were removed from sequencing reads via Cutadapt (v1.2.1) (Martin, 2011). Low-quality reads were trimmed via a sliding-window algorithm in fastp (Chen et al., 2018a). After that, the reads were aligned to the host genome of tobacco via the BMTagger to remove host contamination (Rotmistrovsky and Agarwala, 2011).

Once quality-filtered reads were obtained, taxonomical classification of the metagenomics sequencing reads from each sample was performed via Kraken2 (Wood et al., 2019) against a RefSeq-derived database, which included genomes from archaea, bacteria, viruses, fungi, protozoans, metazoans and viridiplantae. Reads assigned to metazoans or viridiplantae were removed for downstream analysis or Kaiju (Menzel et al., 2016) with greedy-5 mode against a nr-derived database, which included proteins from archaea, bacteria, viruses, fungi, and microbial eukaryotes. Megahit (v1.1.2) (Li et al., 2015) was used to assemble each sample via meta-large preset parameters. The generated contigs (longer than 200 bp) were then pooled together and clustered via mmseqs2 (Steinegger and Söding, 2017) in “easy-linclust” mode, setting the sequence identity threshold to 0.95 and covering the residues of the shorter contig to 90%. The lowest common ancestor taxonomy of the nonredundant contigs was obtained by alignment against the NCBI-nt database via mmseqs2 (Steinegger and Söding, 2017) in “taxonomy” mode, and contigs assigned to Viridiplantae or Metazoa were excluded from the following analysis. MetaGeneMark was used to predict the genes in the contigs. The CDSs of all samples were clustered by mmseqs2 (Steinegger and Söding, 2017) in “easy-cluster” mode, setting the protein sequence identity threshold to 0.90 and covering residues of the shorter contig to 90%. To assess the abundances of these genes, the high-quality reads from each sample were mapped onto the predicted gene sequences via salmon (Patro et al., 2015) in the quasimapping-based mode with “--meta--minScoreFraction = 0.55”, and the CPM (copy per kilobase per million mapped reads) was used to normalize the abundance values in the metagenomes. The functionality of the nonredundant genes was obtained via annotation via mmseq2 (Steinegger and Söding, 2017) in “search” mode against the protein databases of the KEGG, EggNOG, and CAZy databases. EggNOG and GO data were obtained via EggNOG-mapper (v2) (Cantalapiedra et al., 2021). GO ontologies were obtained via map2slim (www.metacpan.org). KOs were obtained via KOBAS (Bu et al., 2021). To predict bacterial functions, high-quality 16S rRNA gene sequences were analyzed using PICRUSt (Phylogenetic Investigation of Communities by Reconstruction of Unobserved States) (Langille et al., 2013).

LEfSe (linear discriminant analysis effect size) was performed to detect differentially abundant taxa and functions across groups on the basis of the taxonomic and functional profiles of nonredundant genes via default parameters (Segata et al., 2011). Beta diversity analysis was performed to investigate the compositional and functional variation in microbial communities across samples via Bray‒Curtis distance metrics (Bray and Curtis, 1957) and visualized via principal coordinate analysis (PCoA), nonmetric multidimensional scaling (NMDS) and the unweighted pair‒group method with arithmetic means (UPGMA) hierarchical clustering (Ramette, 2007).

### 2.4 Correction analysis between metagenomics and metabolites

Multivariate analysis was employed to examine significant differences among microorganisms and metabolites via SIMCA software (version 14.0) and was based on partial least squares discriminant analysis (PLS-DA), PCA, and VIP techniques. PLS-DA is a supervised multivariate statistical analysis technique that combines metabolite changes with experimental grouping information through regression models while reducing dimensions. To assess the model’s quality, we utilize metrics such as R2X, R2Y, and Q2. R2X and R2Y indicate the percentage of variance in the X and Y matrices explained by the PLS-DA model, respectively. Q2, which is calculated through cross-validation, is used to evaluate the predictive ability of the PLS-DA model. Typically, R2 and Q2 values above 0.5 suggest a well-fitted model; however, values slightly below 0.5 can be acceptable, indicating a weaker model. However, supervised classification models such as PLS-DA can be prone to overfitting. To address this, we conduct permutation tests to assess overfitting. If the regression line of Q2 intersects the y-axis at a point less than zero, it indicates the absence of overfitting, indicating the reliability of the PLS-DA model. In addition, correlation analysis was carried out via SPSS software (version 25.0). To obtain insight into the general community structure, OmicsMart (http://www. Omics mart. com) was used for PCoA and variation partition analysis (VPA). A heatmap was generated, and correlation analysis was performed via ChiPlot (https://chiplot.online/). Gephi software (version 0.10.1) was used for visual network analysis (Bastian et al., 2009).

### 2.5 Strain screening and identification

One gram of each FCT sample (A0--A4) was diluted with sterile water, spread on LB agar plates (LB, tryptone 10 g·L-1, yeast extract 5 g·L-1, NaCl 10 g·L-1, agar, 10 g·L-1) and incubated at 37°C for strain isolation. The 16S rRNA gene of each strain was amplified via the primers Bact-27F (5′-AGAGTTTGATCMTGGCTCAG-3′) and Bact-1492R (5′-GGT​TAC​CTT​GTT​ACG​ACT​T-3′) and sequenced. The isolated and purified strains were spot inoculated onto specific screening media for amylase, protease, and cellulase activity. The composition of the media was as follows: amylase screening medium: peptone, 10.0 g; soluble starch, 2.0 g; beef extract, 5.0 g; NaCl, 5.0 g; and agar, 20.0 g, pH 7.0–7.2. Protease screening media included the following: casein (10.0 g), yeast extract (5.0 g), NaCl (4.0 g), and agar (20.0 g), pH 7.2. The cellulase screening media used were KNO_3_ (2.0 g), MgSO_4_ (0.5 g), KH_2_PO_4_ (1.0 g), NaCl (1.0 g), Na_2_HPO_4_ (1.0 g), sodium carboxymethyl cellulose (CMC-Na) (20.0 g), Congo red (0.2 g), and agar (20.0 g), pH 7.0–7.2. The inoculated plates were incubated at 37°C for 48 h. The enzymatic activities of amylase, protease, and cellulase were evaluated on the basis of the ratio of the diameter of the hydrolysis zone (D) to the diameter of the colony (d) (D/d). The degradation index (I) was calculated via the formula used to screen strains with strong macromolecular degradation capabilities (Inan Bektas et al., 2023).

### 2.6 Compound microbial agent preparation and FCT inoculation experiment

The four *Bacillus* strains with the highest amylase, protease, and cellulase activities were functionally screened from the surface of FCT samples. These strains were identified as *Bacillus altitudinis* YS193 (16S rDNA accession No. OP341364), *Bacillus pumilus* YH186 (OP476391), *Bacillus tequilensis* YS154 (OP476354), and *Bacillus velezensis* YS157 (OP476357). Different combinations of strains (Table 1) were inoculated onto the surface of FCTs via the following method. The four strains employed were individually cultivated in 2.4 L of LB medium until they reached the logarithmic growth phase. The cells were mixed and withdrawn via centrifugation, resuspended in 12 L of sterile water, and used as inoculants. The cells were then withdrawn and washed two times with ddH_2_O and inoculated into FCTs at 2% inoculum (v/m) at a cell density of approximately 1 × 10^9^ cfu/mL. The entire fermentation process was carried out at 28°C and 70% RH for 15 days. FCTs inoculated with sterile water were used as controls. All the experiments were repeated three times, each in triplicate.

**TABLE 1:** Compound microbial agent formula and coordination index of tobacco leaf fermentation.

Strain and index	Combination	Nitrogen/Nicotine	Potassium/Chlorine	Total sugar/Nicotine
Group				
CK	sterile water	0.91 ab	3.22 c	7.81 e
T1	YS193+YS157	0.94 a	4.09 b	9.06 bc
T2	YS193+YS154	0.92 a	4.14 ab	9.24 b
T3	YS193+YH186	0.89 ab	3.29 c	8.73 d
T4	YS154+YS157	0.89 ab	3.38 c	8.52 d
T5	YH186+YS157	0.89 ab	4.02 b	8.42 d
T6	YH186+YS154	0.89 ab	4.37 a	8.96 c
C1	YS193+YS154+YS157	0.98 a	4.07 b	9.2 b
C2	YS193+YH186+YS157	0.90 ab	4.07 b	9.06 bc
C3	YS193+YH186+YS154	0.88 b	4.19 ab	9.22 b
C4	YH186+YS154+YS157	0.86 b	3.98 b	9.27 b
F	YS193+YH186+YS154+YS157	0.93 a	4.13 ab	9.67 a

Note: Unique lowercase letters within the same column indicate significant differences between different tobacco varieties, *P* < 0.05.

### 2.7 Determination of the physicochemical components of FCT

The tobacco industry has established six routine testing indicators, including total sugars, reducing sugars, total nitrogen, alkaloids, chlorine, and potassium, to assess the basic composition and quality characteristics of tobacco leaves (Peng et al., 2022b). To investigate the effects of combined microbial inoculants on FCT fermentation, we measured these six indicators. Tobacco leaf samples treated with the combined microbial inoculants were ground via a grinding machine and sieved through a 40-mesh screen to obtain samples for subsequent physicochemical analysis. The total alkaloid content was determined via the continuous flow method according to YC/T 160–2002; the potassium ion content was measured following YC/T 217–2007; the chloride ion content was determined according to YC/T 162–2011; the total nitrogen content was measured following YC/T 161–2002; the water-soluble total sugar and reducing sugar contents were analyzed according to YC/T 159–2019; the starch content in tobacco leaves was determined via the iodine colorimetric method; and the cellulose content was measured via the anthrone colorimetric method. In addition, the quality of tobacco leaves is not solely determined by the absolute content of certain components but also by the relative proportions and harmonious balance among various related components. We calculated the sugar-to-nicotine ratio on the basis of the ratio of total sugar content to nicotine content, the nitrogen-to-nicotine ratio on the basis of the ratio of total nitrogen content to nicotine content, and the potassium-to-chloride ratio on the basis of the ratio of potassium to chloride. The ideal range for the sugar-to-nicotine ratio is typically 8–12. This ratio is closely related to the nicotine content and smoke intensity and thus should not be too low, with an optimal value of approximately 10. It serves as an important indicator for evaluating the smoking flavor and irritancy of tobacco leaves. A ratio below 5 indicates high irritancy, whereas a ratio exceeding 15 suggests insufficient physiological strength. The nitrogen-to-nicotine ratio, which is the ratio of total nitrogen content to nicotine content in tobacco leaves, is ideally close to or slightly less than 1. The potassium-to-chloride ratio, which represents the ratio of potassium to chloride in tobacco leaves, is generally greater than 4 (Qiao et al., 2024; Su et al., 2024; Zhang et al., 2024d).

### 2.8 Sensory evaluation of FCT

We balanced the moisture content of FCT treated with different combinations of *Bacillus* strains and then cut it into shreds to produce cigarettes. The sensory evaluation experiment was conducted at the Technology Center of China Tobacco Anhui Industrial Corporation. The process strictly adhered to the “Sensory Evaluation Methods for Tobacco in Process” (YC/T 415–2011) standard, and a comprehensive assessment of the sensory characteristics of the cigarette products was performed. The evaluation criteria primarily include three categories: aroma, smoke, and taste. Aroma indicators include aroma quality (the overall characteristics and sensory pleasure of the scent emitted during smoking), aroma quantity (the intensity of the fragrance released during smoking), and off-flavors (any additional scents or gases in addition to the primary aroma). Smoke indicators include strength (the perceived intensity of the smoke), concentration (the density or thickness of the smoke), and smoothness (the texture or smoothness of the smoke when inhaled). The taste indicators include irritation (any discomfort in the throat or mouth during smoking), dryness (the sensation of dryness in the mouth or throat caused by smoking), aftertaste (the residual flavor in the mouth after smoking), and sweetness (the perceived level of sweetness in the smoke). Each indicator is scored out of a maximum of 9 points. On the basis of previous research by China Tobacco Anhui Industrial Corporation, the total sensory evaluation score is calculated as follows: except for strength and concentration, each indicator is assigned a specific weight. The formula for the total sensory score (T) is as follows: T = (Aroma Quality × 0.2 + Aroma Quantity × 0.2 + Off-Odor × 0.1 + Smoothness × 0.05 + Irritation × 0.1 + Dryness × 0.05 + Aftertaste × 0.15 + Sweetness × 0.15) × 100/9.

### 2.9 Determination of flavor components in FCT

The flavor compounds in FCT were extracted via simultaneous distillation extraction on a Likens–Nickerson apparatus following Peng et al. (2004c) and analyzed via gas chromatography‒mass spectrometry (GC‒MS, Agilent, Palo Alto, CA, United States) equipped with an HP‒5 MS column (30 m × 0.25 mm×0.25 μm). Pure helium was used as the carrier gas at a flow rate of 20 mL per minute following Gong et al. (2023). For temperature control, the initial temperature was set at 40°C and maintained for 3 min. The temperature was subsequently raised to 160°C at a rate of 15°C per minute. The temperature was then raised to 260°C at a rate of 9°C per minute and held for 1 min. For the mass spectrum, the electron energy was set at 70 eV. The ion source temperature was set at 260°C, and the interface temperature was maintained at 280°C. The scanning mode was full scan from 35 to 350 m/z. The total ion chromatograms of the volatile substances were retrieved from the NIST 14 spectrum library. Qualitative analysis was carried out together with reference documents, and semiquantitative calculations were carried out for each component on the basis of the concentration of the internal standard. Content of volatile components (μg·L^−1^) = concentration of internal standard × three × peak area of component/peak area of internal standard.

### 2.10 Statistical analysis

Spearman’s pairwise correlations were calculated simultaneously via the corr.test function with the psych package in R (version R-3.5.1) to analyze the significance of the correlations. Significantly (*P* value <0.05) high correlations (|*ρ*|>0.6) were visualized via Cytoscape (version 3.6.1).

## 3 Results

### 3.1 Characteristics of metabolites in FCTs during aging

**FIGURE 1:**
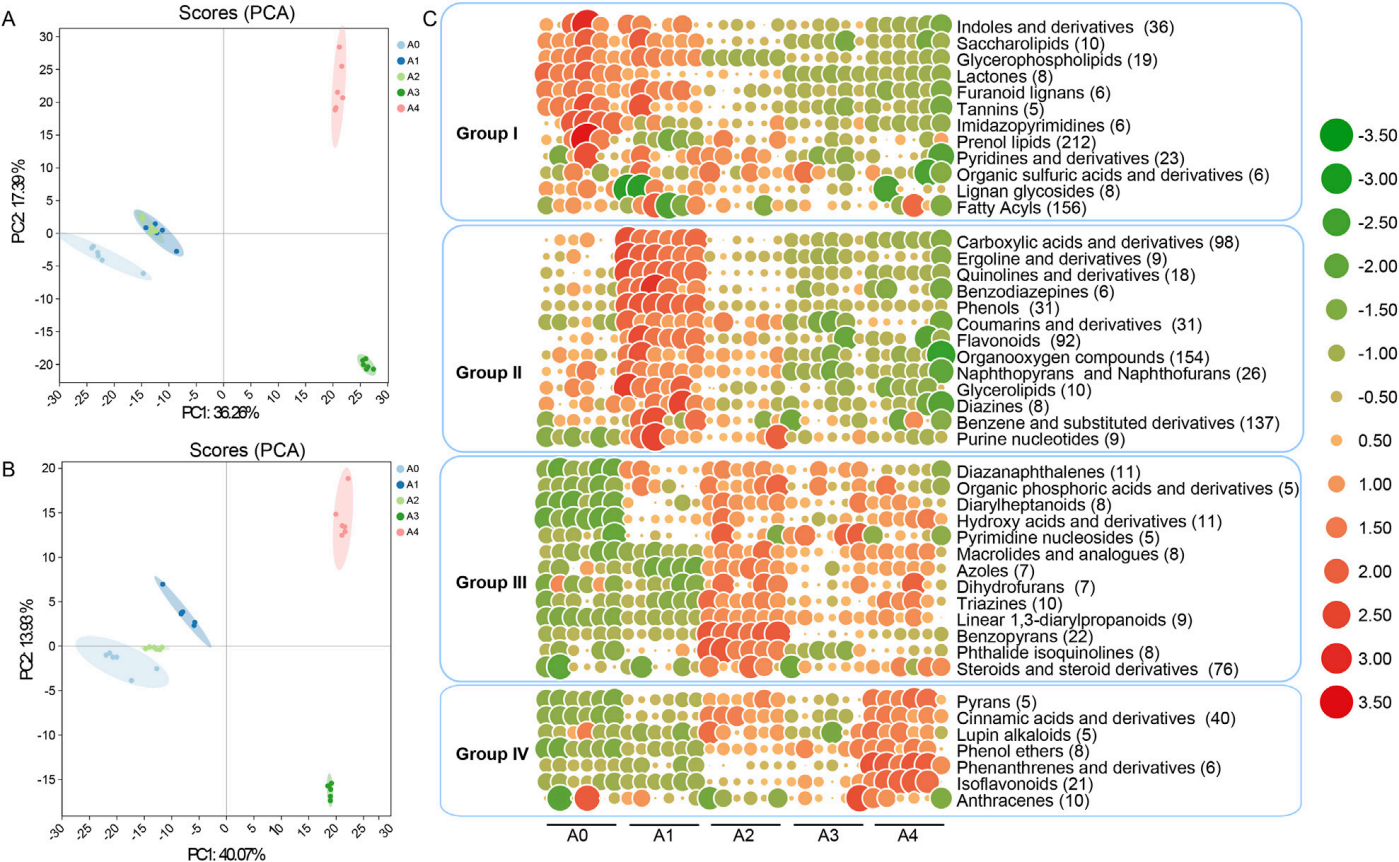
A PCA score map and volcano diagram of tobacco metabolite expression were derived from the UHPLC-Q-TOF MS metabolite spectrum of tobacco **(A)** Positive ion mode (ESI+) and **(B)** negative ion mode (ESI) for the overall sample. The elliptical curve represents the 95% confidence interval **(C)** Heatmap of changes in different categories of metabolic substances during the fermentation process of FCT over different years. The larger the circle and the redder the color are, the more metabolites are present; conversely, the larger the circle and the greener the color are, the fewer metabolites are present. The numbers in parentheses indicate the count of metabolic substances. A1--A4, FCTs aged 0–4 years.

A total of 1781 metabolites were identified from FCT samples aged for different durations, with 1,072 from positive ion mode and 709 from negative ion mode (Supplementary Appendix S1). After these metabolites were categorized into superclasses, they were divided into 12 groups (Supplementary Appendix S1). Among these, lipids and lipid-like molecules comprised 27.12% of the identified metabolites, followed by phenylpropanoids and polyketides (13.81%), organoheterocyclic compounds (13.36%), undefined compounds (12.24%), and benzenoids (11.73%). To observe the metabolite variability of FCT with different fermentation durations, PCA was carried out to explore the inter- and intragroup relationships of the overall samples (Figure 1A). At the 95% confidence level, almost all samples were observed, with parallel samples from the same group clustered together, suggesting that the obtained results were reliable. To better understand the differences in metabolites among groups in FCT samples, OPLS-DA was performed to exclude data unrelated to categorical variables and improve the interpretability of the model to obtain a more reliable model. The results revealed that the FCT samples were completely separated and clustered together according to fermentation time in the horizontal direction with 95% confidence (Supplementary Figure S1). A volcano diagram based on the fold change (FC) analysis (FC > 1.5 or <0.67, p value <0.05) revealed that the FCT aging process was characterized by the metabolism, transformation, and synthesis of alkaloids, their derivatives, and benzene ring compounds (Supplementary Figure S2).

A more detailed classification of the metabolites was performed to observe the changes in different types of metabolic substances during FCT fermentation over different years (Supplementary Appendix S1). The metabolites were divided into 125 classes, with 45 classes containing more than 5 substances in each class. Prenol lipids were the most abundant, with 212 metabolites, followed by fatty acyls (156), organooxygen compounds (154), and benzene and substituted derivatives (137). The substances exhibited different trends as the number of years of FCT fermentation increased. Overall, they can be categorized into four types. For Group I, during the first year of FCT fermentation, most substances did not change significantly compared with those in fresh tobacco. However, the content of Prenol lipids (212) significantly decreased during the first year of fermentation. The substances whose contents significantly decreased in the second year primarily included fatty acyls (156), indoles and derivatives (36), and glycerophospholipids (19), among others. For Group II, the metabolic substances rapidly accumulated during the first year of FCT fermentation and peaked by the end of the first year, after which they began to decline during the second year. This group includes mainly organooxygen compounds (154), benzene and substituted derivatives (137), flavonoids (92), and carboxylic acids and derivatives (98). For Group III, the metabolic substances began to accumulate either in the first or second year of FCT fermentation and peaked in the second year, after which they gradually decreased or remained stable. This group includes mainly steroids and steroid derivatives (76), benzopyrans (22), and hydroxy acids and derivatives (11). For Group IV, similar to Group III, these substances gradually increased in the second year of fermentation but reached their peak abundance in the fourth year. This group includes mainly cinnamic acids and derivatives (40), isoflavonoids (21), and anthracenes (10). Overall, the trend of metabolite changes became slower after the second year of fermentation with FCT. The differentially abundant metabolites among the groups were identified on the basis of a VIP value >1.0 and a *p* value <0.05 (Supplementary Table S1). During the first year of FCT fermentation, an increase in several intermediate metabolites was observed. For example, there was a significant increase in the concentrations of cyclic AMP (group II), proline (group II), phenylacetaldehyde (group II), quinic acid (group II), threonic acid (group II), quercetin (group II), L-malic acid (group III), and tryptophan (group III). In comparison, during the second year of fermentation, color or flavor substances such as quercetin (group II), maleic acid (group II), phenylalanine (group II), L-malic acid (group III), gulonic acid (group III) and alkaloids (group IV) increased and became stable in the following years. Moreover, the concentrations of tryptamine (group I), tryptophan (group I), proline (group II), and trehalose (group II) decreased significantly (Supplementary Figures S3,S4). Overall, the metabolites became stable during the second year and subsequent years of FCT fermentation, indicating that most of the fermentation process was completed in the second year (Figure 1B; Supplementary Figures S3,S4).

### 3.2 Microbial diversity on the surface of FCT

**FIGURE 2:**
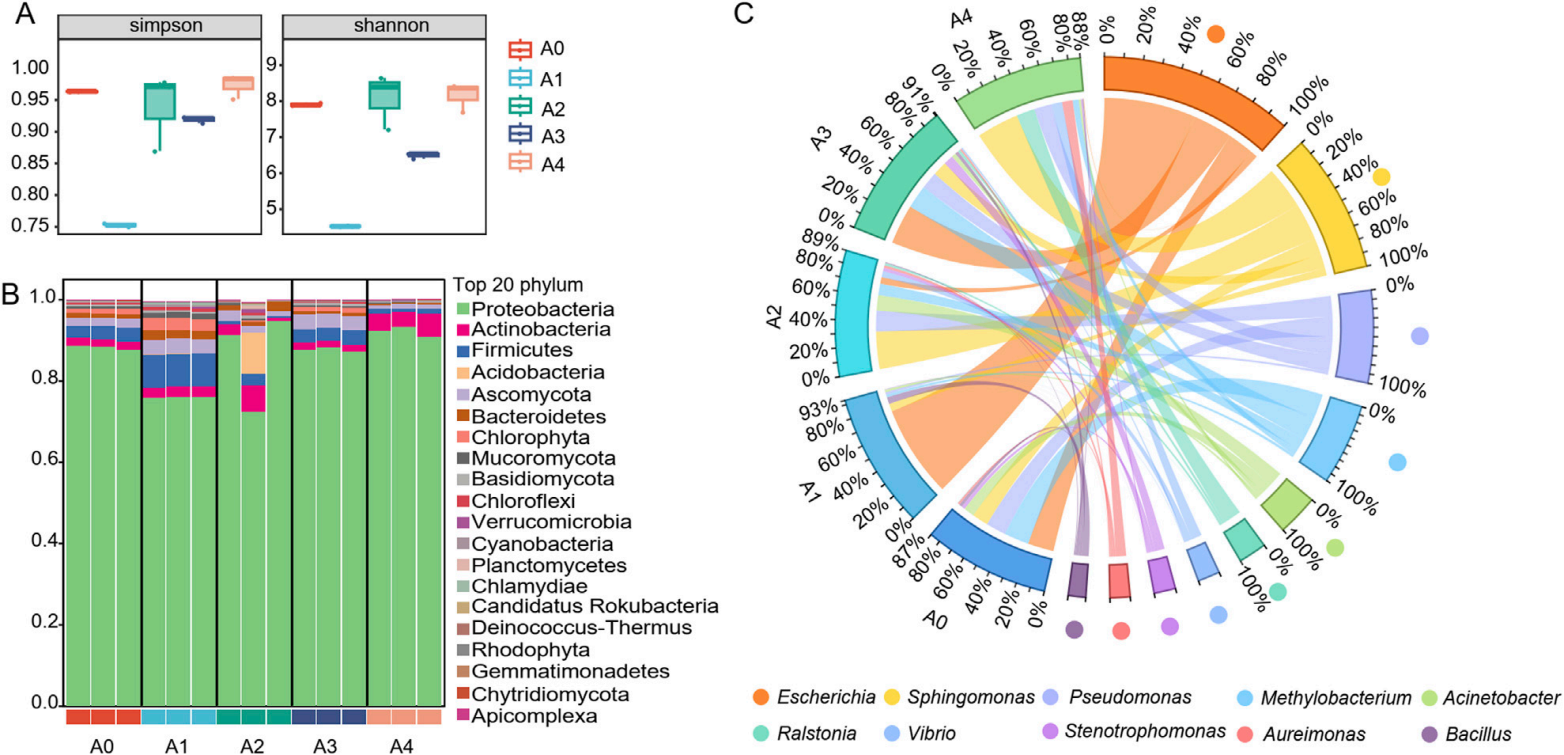
The diversity of the microbial community in tobacco. **(A)** The relative abundances of microbial communities at the phylum level **(B)** and genus level **(C)** are shown in stacked plots, with each bar representing the average relative abundance of the top 20 microbiomes in the sample. A1-A4, FCTs aged 0–4 years.

Five groups of FCT samples collected from different fermentation years were employed for metagenomic analysis. The results revealed that the coverage of all the samples was greater than 99%. The α diversity of the bacterial communities in the FCT samples was assessed via Simpson diversity and Shannon diversity indices. The aging process was found to have a strong effect on the α diversity of the FCT microbial community. After 1 year of fermentation in FCT, the sample abundance significantly decreased. In comparison, the richness and evenness of the sample communities gradually increased after 2 years of fermentation (Figure 2A).

To better understand the differences between the microbial communities of FCTs aged for different durations, beta diversity was explored via PCoA via the Bray‒Curtis distance algorithm (Supplementary Figure S5). The PCoA results suggested that the contribution rates of principal coordinates PC1 and PC2 were 55.31% and 13.43%, respectively, indicating that these two principal coordinates can explain most of the samples. The aggregation of samples within the same group indicates reliable reproducibility of the samples. In this case, as the aging process progressed, the distances between the samples at different aging stages dispersed rather than clustered together, suggesting that the microbial community composition in the FCT changed with the aging process.

The dominant phyla of microbes, including Proteobacteria, Actinobacteria, Firmicutes, Ascomycota, Bacteroidetes, and Chlorophyta, were identified from all five samples, suggesting the consistent presence of these microbial groups across various stages of FCT maturation (Figure 2B). Compared with that in fresh tobacco leaves (A0), the abundance of Proteobacteria in FCT-fermented tobacco for the first year (A1) decreased, whereas the abundances of Firmicutes, Ascomycota, Bacteroidetes, and Chlorophyta increased. The FCT fermented for 2 years (A2) resulted in complex changes in the abundance of microbial communities at the phylum level, with the abundance of Proteobacteria and Actinobacteria increasing while the abundance of Ascomycota, Bacteroidetes, and Firmicutes decreasing. After 3 years of FCT fermentation (A3), the abundance of Ascomycota still increased compared with that after 1 year of fermentation, but the abundances of the other phyla were similar to those of fresh tobacco leaves (A0). After 4 years of FCT fermentation (A4), the microbial abundances of Proteobacteria and Actinobacteria increased, whereas the abundances of Firmicutes, Ascomycota, Bacteroidetes, and Chlorophyta decreased (Figure 2B).

At the genus level, the top 10 dominant genera were *Escherichia*, *Sphingomonas*, *Pseudomonas*, *Methylorubrum*, *Acinetobacter*, *Ralstonia*, *Vibrio*, *Stenotrophomonas*, *Aureimonas*, and *Bacillus* (Figure 2C). In the fresh tobacco leaf samples (A0), *Escherichia*, *Methylobacterium*, *Pseudomonas*, and *Sphingomonas* accounted for relatively high proportions, all exceeding 10%. After 1 year of fermentation with FCT (A1), the proportion of *Escherichia* significantly increased from 16.5% to 58.5%. Additionally, *Bacillus* increased from 2.0% in fresh tobacco leaves to 4.3%. Similarly, (A2), the proportion of *Escherichia* decreased from 58.5% to 4.2%. However, the proportions of *Sphingomonas* (4.9%–23.7%), *Pseudomonas* (1.6%–13.1%), *Acinetobacter* (1.5%–9.6%), and *Methylobacterium* (1.8%–7.3%) significantly increased. After 3 years of FCT fermentation (A3), the proportion of *Escherichia* rose to 25.2%, and that of *Methylobacterium* increased to 14.7%. After 4 years of fermentation with FCT (A4), the proportion of *Escherichia* decreased again to 0.33%, whereas *Sphingomonas* (26.8%), *Ralstonia* (11.7%), and *Pseudomonas* (11.2%) were the dominant bacteria, with proportions greater than 10%. Overall, the dominant bacterial genera in FCT undergo irregular changes at different stages of fermentation, indicating that different bacteria may be required to metabolize the metabolic products at each stage of fermentation.

### 3.3 Functional annotation of the microbial community

**FIGURE 3:**
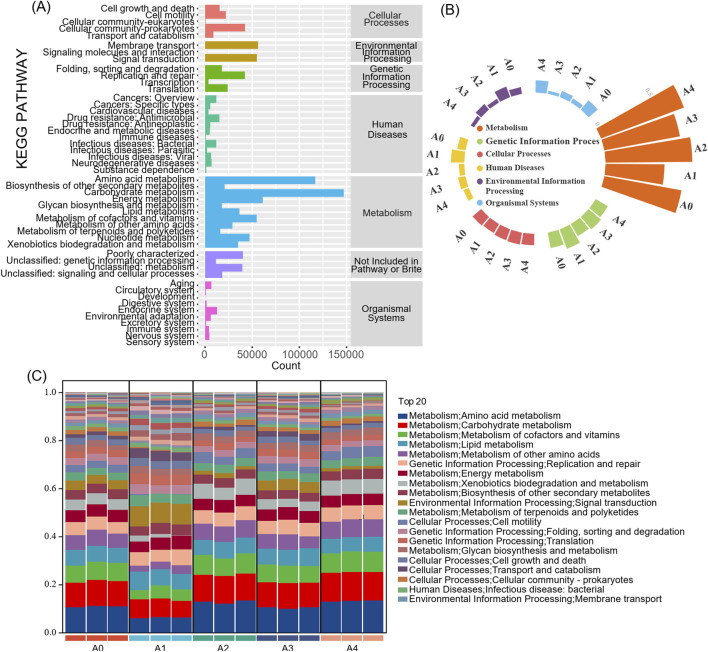
The abundance of KEGG metabolic pathways of the microbial community in flue-cured tobacco samples **(A)** KEGG metabolism of the whole sample; **(B)** Histogram of KEGG primary metabolic pathways; **(C)** KEGG metabolism at the secondary level of tobacco samples in different years. A1-A4, FCTs aged 0–4 years.

The bacterial community sequencing data from the FCT samples were analyzed via PICRUSt to predict the potential functions of the microbial communities in the samples (Figure 3A). The results revealed that the KEGG pathway richness of all the samples was similar and that all the samples involved six types of metabolic pathways in the primary functional layer (Figure 3B). These pathways are metabolism (59.1%), environmental information processing (11.3%), cellular processes (9.1%), genetic information processing (8.9%), human diseases (7.7%), and organismal systems (3.8%). Among them, metabolism had the highest percentage in each sample, indicating that microorganisms on the surface of FCT participate in the aging process of FCT mainly through metabolic pathways.

The secondary functional layer of the predicted genes was further analyzed. The top 20 subfunctional layers, along with their respective abundances, are presented in Figure 3C. The results indicated that the top 20 subfunctions collectively accounted for over 87% of the total samples. Among these, the metabolic function of the primary functional layer accounted for 60%. Taken together, amino acid metabolism, carbohydrate metabolism, the metabolism of cofactors and vitamins, lipid metabolism, and the metabolism of other amino acids have high gene abundances. Compared with fresh tobacco leaves (A0), there was a significant reduction in the enrichment of genes related to amino acid metabolism, carbohydrate metabolism, metabolism of cofactors and vitamins, and xenobiotic biodegradation and metabolism pathways during the first year of FCT fermentation (A1). However, the proportion of these genes recovered from the second year of fermentation. In contrast, during the first year of FCT fermentation, genes associated with signal transduction; the metabolism of terpenoids and polyketides; folding, sorting and degradation; translation; cell growth and death; and transport and catabolism were enriched, accounting for the highest proportion among the five sample groups. These results indicate that although microbial abundance decreases in the first year of FCT fermentation, the microbes remain active and carry out important succession activities. From the second year of FCT fermentation onward, these microbes begin to increase their metabolism of amino acids and carbohydrates.

### 3.4 Correlation analysis of tobacco microorganisms and substance metabolism during fermentation

**FIGURE 4:**
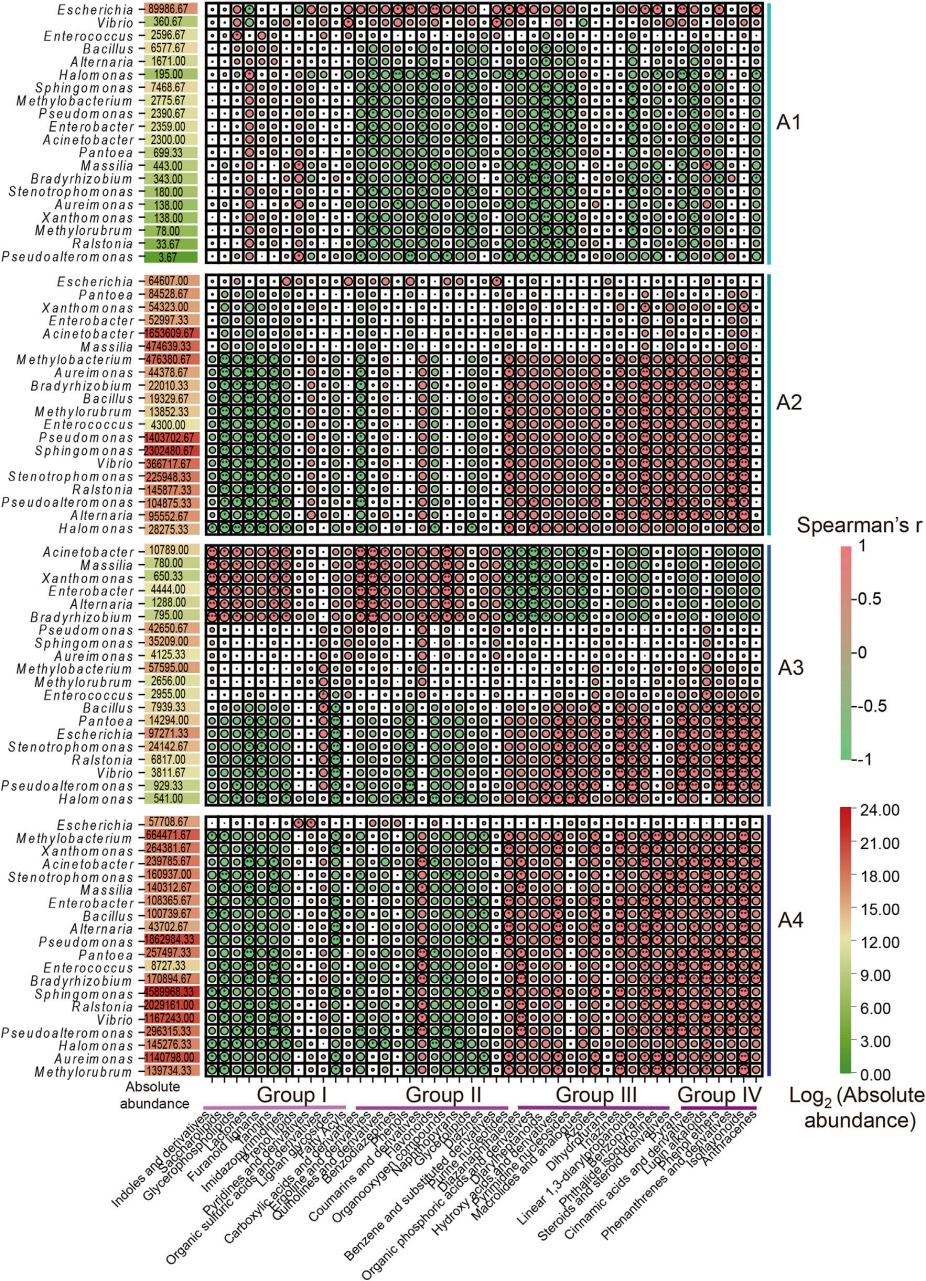
Spearman correlation analysis between the absolute abundance of the top 20 microbial genera in FCT fermentation samples and metabolic substances across different groups. A1-A4, FCTs aged 0–4 years.

**FIGURE 5:**
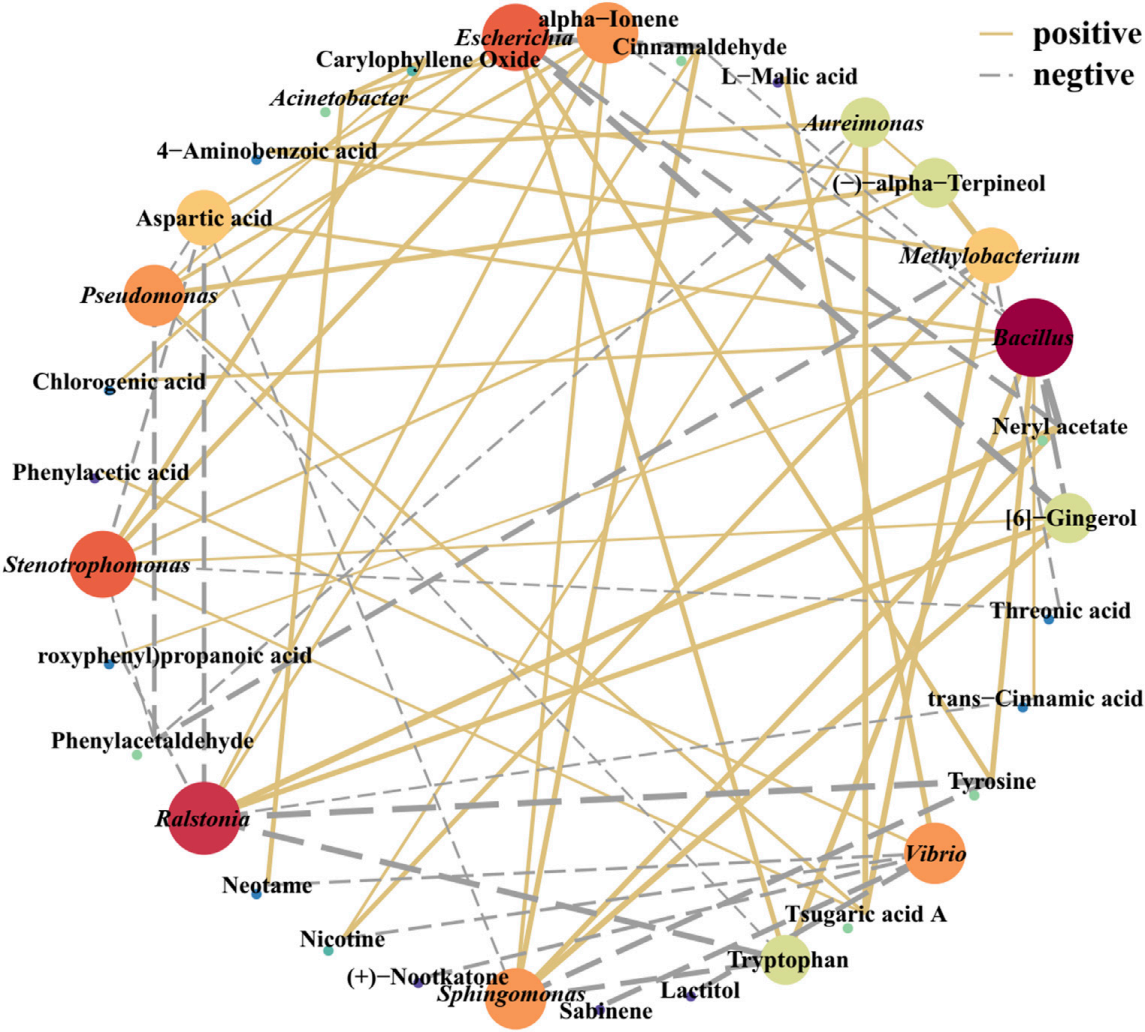
Correlation network analysis of the microbial community and volatile components. The red solid line indicates a positive correlation, and the blue dotted line indicates a negative correlation. The line thickness indicates the correlation strength; the point size/color depth indicates the number of related objects.

The correlation between metabolic substances and microbial community (top 20) abundance in FCT samples was explored via a Spearman rank correlation coefficient matrix (Figures 4, 5; Supplementary Figure S6). We found that the correlations between microbial communities and metabolites, analyzed based on relative abundance and absolute abundance respectively, exhibited inconsistent results (Supplementary Figure S6; Figure 4). By using microbial absolute abundance for correlation analysis, the relationship between microbial succession and metabolite concentration changes could be more accurately determined. During the first year of FCT fermentation, *Escherichia* and *Vibrio* showed positive correlations with the metabolites of groups I–IV. Eighteen other bacterial genera, including *Bacillus*, *Enterococcus*, and *Alternaria*, exhibited positive correlations with the metabolites of group I but negative correlations with those of groups II–IV (Figure 4). Notably, the metabolites of group I accumulated most significantly in the first year (Figure 1), indicating that these metabolites were primarily contributed by the metabolic activities of the 20 bacterial genera mentioned above. During the second year of fermentation, 19 genera including *Methylobacterium* and *Bacillus* predominantly showed negative correlations with the metabolites of groups I–II, but positive correlations with those of groups III–IV (Figure 4). However, the metabolites of group II increased significantly in the second year (Figure 1), and *Escherichia* was the main genus exhibiting positive correlations with most substances in group II (Figure 4). This indicates that *Escherichia* made the greatest contribution to the metabolites of group II in the second year. During the third year, eight genera including *Bacillus*, *Pantoea*, and *Stenotrophomonas* continued to exhibit positive correlations with metabolites of groups III–IV. Conversely, six genera such as *Acinetobacter* and *Massilia* showed positive correlations with metabolites of groups I–II but negative correlations with those of groups III–IV (Figure 4). Notably, groups III–IV accumulated higher levels of metabolites in the third year (Figure 1), which were primarily contributed by the metabolic activities of the eight genera including *Bacillus*.In the fourth year, the substances mainly accumulated in group IV (Figure 1), and the top 20 abundant genera all showed positive correlations with it (Figure 4). These results indicate that these core microbial communities play crucial roles in metabolic processes during FCT fermentation.

Volatile flavor is an important parameter of cigarette quality. The correlations between volatile flavors in tobacco leaves and surface microbial communities were analyzed via Cytoscape software and visualized via a network diagram (Figure 5). *Bacillus*, *Escherichia*, *Ralstonia*, *Stenotrophomonas*, *Pseudomonas, Sphingomonas*, *Vibrio*, and *Aureimonas* were significantly correlated with these various flavor substances (|r| >0.6; *P* < 0.05). Among them, *Pseudomonas* was positively correlated with terpineol, whereas *Ralstonia* was positively correlated with cinnamaldehyde and neryl acetate (Appendix S1). Additionally, *Bacillus* and *Escherichia* were negatively correlated with bitter and spicy substances, such as gingerol, which are unpleasant. Conversely, *Bacillus* and *Escherichia* were positively correlated with amino acids and other flavor-promoting substances. For example, *Bacillus* is positively correlated with amino acids (tryptophan, tyrosine, aspartate acid, phenylalanine), aromatic compounds (phenylacetaldehyde, phenol, benzyl alcohol, and coumarin) and other substances (Supplementary Table S2). These results suggested that *Bacillus* and *Escherichia* may play a role in enhancing the presence of these compounds during fermentation. The presence of core microbial communities and their correlation with these flavor-enhancing substances indicate their involvement in shaping the unique flavor characteristics of the final tobacco product.

### 3.5 The impact of combined microbial inoculants on the physicochemical components of FCT

**FIGURE 6:**
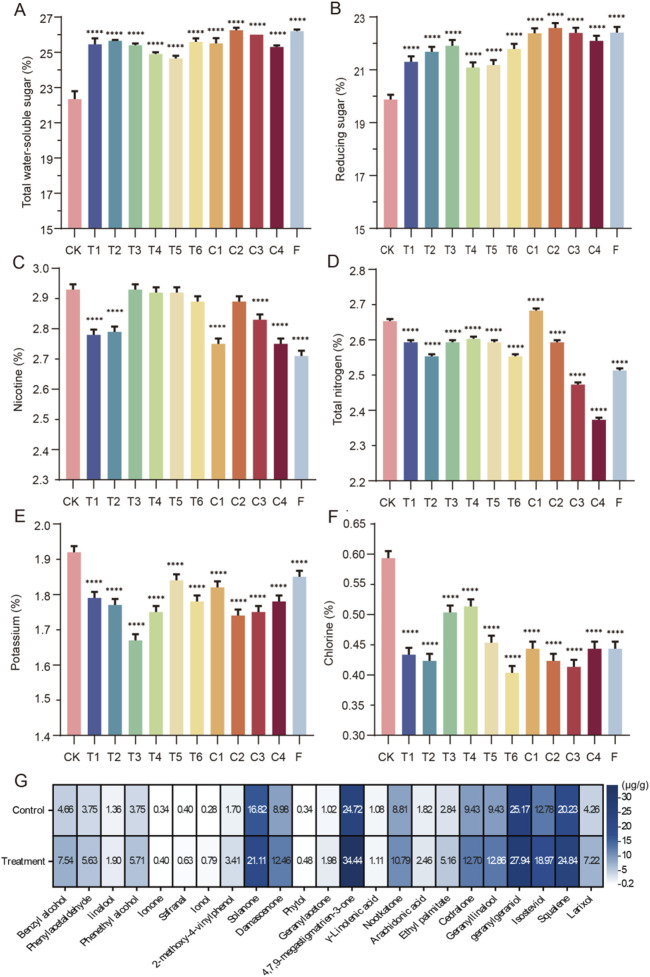
The quality indicators of FCT after fermentation with different microbial combinations **(A)** and **(B)** Sugar content during tobacco leaf fermentation; **(C)** and **(D)** content of nitrogen substances during tobacco leaf fermentation; **(E)** and **(F)** inorganic ion content during tobacco leaf fermentation; **(G)** heatmaps illustrating the content of volatile fragrance-causing components per gram in FCT samples treated with a combination of four *Bacillus* strains for 15 days. For grouping information, refer to Table 1.

A total of 107 bacterial strains were isolated from tobacco leaf samples from different years and identified molecularly via 16S rDNA sequencing (16S rDNA accession numbers: OP341362–OP341371, OP456963–OP456972, OP456334–OP456427; Supplementary Table S3). The isolated microorganisms were predominantly *Bacilli*, with *Bacillus* and *Terribacillus* being the dominant genera, indicating their high activity during tobacco leaf fermentation. Four strains, *Bacillus altitudinis* YS193 (OP341364), *Bacillus pumilus* YH186 (OP476391), *Bacillus tequilensis* YS154 (OP476354), and *Bacillus velezensis* YS157 (OP476357), were screened from the surface of the FCT samples because of their favorable amylase, protease, and cellulase activities (Supplementary Table S3). These strains were subsequently combined into a composite for FCT fermentation. Since the effects of single-strain treatments on FCT were not significant compared with those of the control group (data not shown), we directly present the data for pairwise combinations, triple combinations, and combinations of all four strains (Figure 6).

After bioenhanced fermentation with microbial agents, the sugar content in tobacco leaves significantly increased. Among them, group F (YS193, YH186, YS154, and YS157) and group C2 (YS193, YH186, and YS157) presented the most significant improvement, with the total sugar content increasing by approximately 14.5% and the reduced sugar content increasing by 12.7% (Figures 6A,B). We found that the nicotine content in tobacco leaves treated with microbial combinations from T1 (YS193 and YS157), T2 (YS193 and YS194), C1 (YS193, YS194, and YS157), C3 (YS193, YH186, and YS154), C4 (YH186, YS154, and YS157), and group F (YS193, YH186, YS154, and YS157) was significantly lower than that in the control group, indicating that these combinations effectively reduced the nicotine content in tobacco leaves (Figure 6C). The total nitrogen content in tobacco leaves fermented with different microbial combinations was lower than that in the control group, with the most significant reduction observed in groups C3 (YS193, YH186, and YS154), C4 (YH186, YS154, and YS157), and F (YS193, YH186, YS154, and YS157; Figure 6D). After fermentation with different microbial combinations, the potassium and chlorine contents in FCT decreased but remained within the standard range (0.05%–3% and 1%–3%, Figures 6E,F). By comprehensively calculating the nitrogen-to-nicotine ratio, potassium-to-chlorine ratio, and sugar-to-nicotine ratio, we found that the sugar-to-nicotine ratio of the FCTs in group F was closest to 10, indicating that the FCTs in group F may have the best taste (Table 1).

### 3.6 Sensory evaluation of FCT fermented with functional strain combinations

The sensory evaluation scores of FCTs treated with different *Bacillus* combinations varied. The control group treated with sterile water had the lowest total sensory score (Supplementary Table S4). Compared with those of the other treatments, the sensory evaluation results of group F, which was treated with a composite microbial inoculant, indicated several positive attributes (Supplementary Table S4). Specifically, this group presented high scores for aroma quality, aroma quantity, aftertaste, and sweetness. This suggests that the fermentation process, potentially enhanced by the composite microbial inoculant, effectively promoted the development of desirable aromatic compounds within the leaves or achieved a balanced concentration of these compounds, providing evaluators with a pleasant olfactory experience. Additionally, the treatment effectively reduced the presence of undesirable substances or contaminants in the leaves, improving their overall purity and imparting satisfactory sensory characteristics to the tobacco leaves, resulting in a pleasant aftertaste during consumption.

### 3.7 Content of volatile aroma components in FCT

GC‒MS was used to analyze the volatile components in FCT after 15 days of fermentation, aiming to investigate the impact of the F group of four *Bacillus* combinations on the flavor of FCT. Compared with those of the control FCT samples, the contents of phenylacetaldehyde and other aromatic compounds were significantly greater (Figure 6G). Among them, benzyl alcohol and phenylacetaldehyde are group II substances of benzene and substituted derivatives (Appendix S1). These substances significantly increased after 15 days of treatment with mixed strains, which is consistent with the performance of FCT during the initial fermentation stage in industrial production (A1, Figure 1). The changes in these components may be related to the involvement of *Bacillus* in the metabolic pathway of aromatic amino acids in FCT (Supplementary Figure S7). Phenylalanine, tyrosine, and tryptophan are the precursors of aromatic compounds. By changing the aromatic amino acids in FCT, *Bacillus* improves the aroma substances in FCT, thus significantly improving the aroma characteristics of tobacco and increasing its floral and fruity flavors.

## 4 Discussion

Current studies have shown that the dynamic changes in microorganisms and the metabolites produced during tobacco fermentation have important impacts on the quality of tobacco (Zhang et al., 2024a). However, in actual industrial production processes, the relationship between microbial succession during long-term FCT fermentation and metabolites remains unclear. In this study, the microbial community structure and function of FCT fermented for 0–4 years were analyzed, and the changes in metabolites in tobacco leaves in different years were analyzed. Based on the types of changes in metabolites in different years during FCT fermentation, we classified these metabolites into four groups (Figure 1B). Among them, prenol lipids and fatty acyls, which are basic compounds contained in fresh FCT, belong to group I (Dunkle, et al., 2016) and gradually degrade with increasing years of fermentation. Group II includes intermediate metabolites such as carboxylic acids and derivatives and organooxygen compounds, which reach a peak in the first year of FCT fermentation and then begin to decrease. Flavor substances in groups III and IV, such as cinnamic acids and derivatives (Gunia-Krzyżak et al., 2018), isoflavonoids (Li et al., 2020b), gulonic acid (Quitmann et al., 2013), and alkaloids (Debnath et al., 2018), are closely associated with the flavor characteristics of tobacco. In industrial production processes, based on production experience, FCT fermentation for 2 years is generally sufficient to produce cigarettes. However, the underlying scientific principles remain unclear. Our findings revealed that group III substances reached their highest levels after the second year. Among these, steroids and steroid derivatives were the most abundant categories. Steroids are a subclass of terpenoids biosynthesized from terpene precursors and serve as critical components in food flavor composition (Maswal et al., 2023). Currently, there is no research on the role of benzopyran in food flavor. However, the benzopyran class, particularly, has garnered significant attention in drug development due to its broad spectrum of pharmacological activities, such as antibacterial, antifungal, antiviral, antitumor, antioxidant, and anti-inflammatory properties (Xi et al., 2024). Cinnamic acids and their derivatives, which are crucial flavor compounds (Gunia-Krzyżak et al., 2018), remain at consistently high levels during the second and fourth years of tobacco fermentation (Figure 1B). We also found that the FCT aging process was characterized by the metabolism, transformation, and synthesis of alkaloids and their derivatives, benzene ring compounds, and related compounds (Figure 1B; Supplementary Figures S3,S4). Alkaloids, classified as secondary metabolites of plants, are nitrogen-containing natural bioactive compounds that exhibit higher concentrations during the second and fourth years (Debnath et al., 2018). During the aging stage of FCT, alkaloids and their derivatives can modulate the taste, aroma, and combustion characteristics of tobacco (Xue et al., 2023). However, higher nicotine concentrations do not necessarily equate to superior tobacco leaf quality. In fact, nicotine levels must remain within a specific optimal range, as excessive concentrations have been shown to diminish sensory quality (Carstens and Carstens, 2022). Therefore, although alkaloids reach their peak concentration in the fourth year of FCT fermentation, industrial production still adheres to a 2-year fermentation period. Additionally, benzene compounds (group II) are pivotal in the aging process of tobacco leaves because they are present in many essential chemical components of tobacco, such as aromatic phenols, coumarins, and phenolic acids. These compounds undergo a series of biochemical and chemical changes during tobacco aging, directly impacting the quality and characteristics of the tobacco (Yan et al., 2022). Overall, the metabolites became stable or exhibited minimal changes after the second year of FCT fermentation (Figure 1B; Supplementary Figures S3,S4), indicating that 2 years of fermentation are essential for FCT fermentation, which supports industrial practices in which the fermentation of tobacco typically spans approximately 2 years. In comprehensive consideration, the primary flavor compounds in FCT fermentation accumulate in substantial quantities within 2 years, meeting production requirements. Although certain flavor substances exhibit favorable accumulation by the fourth year, practical production necessitates balancing factors such as time costs. Thus, a 2-year fermentation period for FCT likely represents the optimal temporal choice for tobacco production.

Previous research has indicated that microorganisms can reduce the duration of tobacco fermentation and that various types of microorganisms can modify the eventual flavor of tobacco (Zhang et al., 2024b). The dominant bacterial genera in FCT undergo irregular changes at different stages of fermentation, indicating that different bacteria may be required to metabolize metabolic products at each stage (Figure 2). After 1 year of fermentation in FCT (A1), the sample abundance significantly decreased. In contrast, the richness and evenness of the sample communities gradually increased after 2 years of fermentation (A2, Figure 3A). We speculate that the influence of the fermentation environment and the high-temperature redrying process may favor the proliferation of dominant core microorganisms, consequently diminishing the richness and diversity of the microbial community during the first 2 years of fermentation. The proportion of *Bacillus* significantly increased from 2.0% to 4.3% in the A1 sample group compared with that in the fresh tobacco group (A0), suggesting that *Bacillus* plays important roles in the first year of fermentation and can be used as one of the biomarkers with the greatest change in the microbial communities during the first year of FCT fermentation. During the first year of fermentation, there was a significant increase in intermediate metabolites belonging to group II, whereas the basic substances of tobacco leaves belonging to group I decreased (Figure 1A). Through the combined analysis of metabolomics and the microbiome, we found that *Bacillus* showed trends consistent with these changes, which further supports the importance of *Bacillus* in the first year of fermentation. *Bacillus* has been documented as a prevalent functional microbe in the aging process of tobacco and is pivotal in the fermentation of tobacco (Zhang et al., 2024b; Zhang et al., 2024c). *Bacillus* can promote tobacco fermentation and improve the flavor and quality of tobacco leaves (Ma et al., 2024). For example, Cai et al. reported that *Bacillus* can produce different volatile flavor compounds by breaking down aroma-causing precursors such as carotenoids (Cai et al., 2022). In our study, during the FCT fermentation process, carbon sources such as lactose and trehalose were negatively correlated with the core microbial community (Supplementary Appendix S1). However, the core microbial community was significantly positively correlated with amino acids and other flavor-promoting substances (Figure 5). *Bacillus* is positively correlated with amino acids such as tryptophan, tyrosine, aspartic acid, and phenylalanine. Additionally, it is associated with aromatic compounds and other substances (Figure 5). *Bacillus* promotes the metabolism of these aromatic substances, which can complete the aroma accumulation of the early stage and accelerate the fermentation efficiency of FCT. These results suggest that as FCT fermentation progresses, microbes consume a significant amount of energy, and amino acids play a crucial role in the synthesis of flavor substances, with *Bacillus* playing a key role in this process.

*Bacillus* secretes various enzymes, such as amylases (Chen et al., 2017b), proteases (Ma et al., 2024), and cellulases (Yao et al., 2022), which degrade organic matter. These enzymes play a role in enhancing tobacco sweetness and softness, reducing irritation, and improving aroma and flavor. The total sugar-to-nicotine ratio serves as a critical parameter for evaluating the balance between the flavor and harshness of tobacco leaves. An excessively high ratio diminishes the strength and flavor of smoke, whereas an overly low ratio increases smoke irritation (Zhang et al., 2024d). In China, a sugar-to-nicotine ratio close to 10 is considered optimal. We found that the combination of *Bacillus* strains YS193, YH186, YS154, and YS157, which exhibit high amylase, protease, and cellulase activities, not only reduced the macromolecule content in roasted tobacco but also achieved a sugar-to-nicotine ratio closest to the optimal value of 10 after FCT treatment. The contents of both total sugars and reducing sugars increased. Sugars significantly impact the sensory quality of tobacco, as they generate acids that can attenuate the strong flavor of the smoke and convert it into several aromatic substances through meladic, caramelization, and pyrolysis reactions (Yang et al., 2014). The results of GC‒MS confirmed the increase in aromatic compounds in the FCT samples caused by *Bacillus* (Figure 6). Our research revealed a microbial combination capable of effectively fermenting FCT. Subsequent studies will aim to further optimize fermentation conditions. Additionally, the superiority of this fermentation method needs to be further confirmed by analyzing its effects on a wider variety of tobacco types.

## 5 Conclusion

The metabolites and microbial community of FCT fermented for different durations were analyzed. The FCT aging process is characterized by metabolic, transformational, and synthetic activities involving alkaloids, their derivatives, and benzene ring compounds. Carbohydrate and amino acid metabolism plays a vital role in secondary metabolic pathways. *Escherichia*, *Bacillus*, *Enterococcus*, *Alternaria*, *Vibrio*, and *Halomonas* play core roles in the degradation of basic substances during the first year of FCT fermentation. Moreover, microorganisms such as *Sphingomonas*, *Vibrio*, *Ralstonia*, *Pseudoalteromonas*, and *Halomonas* are key players in the production of flavor compounds from the second to fourth years of fermentation. Carbon sources were negatively correlated with the core microbial community, such as *Bacillus*, whereas amino acids and other flavor-promoting substances were significantly positively correlated. In addition, we screened a combination of four *Bacillus* strains that effectively optimized the sugar-to-nicotine ratio of FCT and increased the levels of volatile compounds associated with the aromatic amino acid metabolic pathway in FCT. This study provides a theoretical foundation for optimizing the fermentation process to produce high-quality tobacco with enhanced aromatic characteristics.

## Data Availability

The datasets presented in this study can be found in online repositories. The names of the repository/repositories and accession number(s) can be found in the article/Supplementary Material.
