# Supplementary material for: Microbial community succession during tobacco fermentation reveals a flavor-improving mechanism
Source: Front Bioeng Biotechnol. 2025 Jul 21;13:1627842. doi: 10.3389/fbioe.2025.1627842 (PMC12319770; doi:10.3389/fbioe.2025.1627842)

Supplementary material

Table. S1 Significant difference metabolites

Comparison group	Name（TOP 5）	VIP (variable importance in the projection) value	m/z	Number of differential metabolites
A0 vs A1	trans-Vaccenic acid	10.384	281.252	169
	RNEHQZRKZJSYOL-UHFFFAOYSA-N	9.046	474.262	
	Tulipanin	7.937	609.149	
	N-Acetyl-DL-tryptophan	6.141	245.093	
	9-Hydroxy-10E,12Z-octadecadienoic acid	5.837	295.226	
A1 vs A2	Tulipanin	11.356	609.149	145
	Cryptochlorogenic acid	8.123	353.088	
	RNEHQZRKZJSYOL-UHFFFAOYSA-N	7.643	474.262	
	N-Acetyl-DL-tryptophan	6.450	245.093	
	MXFUJXIOAUPXPT-LAGVYOHYSA-N	6.363	387.182	
A2 vs A3	Erucamide	15.131	338.345	129
	Kaempferol-3-O-rutinoside	9.254	593.153	
	MXFUJXIOAUPXPT-LAGVYOHYSA-N	7.845	387.182	
	13'-Hydroxy-alpha-tocopherol	5.958	429.373	
	Cryptochlorogenic acid	5.912	353.088	
A3 vs A4	Kaempferol-3-O-rutinoside	11.130	593.153	112
	13'-Hydroxy-alpha-tocopherol	9.353	429.373	
	Caffeic acid hexoside	7.854	341.085	
	Chlorogenic acid	5.611	353.089	
	Quinic acid	5.406	191.056	

Table. S2 Correlation data between *bacillus* and metabolites in tobacco leaves

Data1	Data2	rho	p-value	relation
Bacillus	Tryptophan	0.916667	0.000507	positive
Bacillus	Honokiol	0.883333	0.001591	positive
Bacillus	Tyrosine	0.883333	0.001591	positive
Bacillus	Aspartic acid	0.766667	0.015944	positive
Bacillus	Chlorogenic acid	0.733333	0.024554	positive
Bacillus	trans-Cinnamic acid	0.716667	0.029818	positive
Bacillus	3-(2-Hydroxyphenyl)propanoic acid	0.666667	0.049867	positive
Bacillus	Phenylacetaldehyde	0.466667	0.205386	positive

Bacillus	Sabinene	0.45	0.224216	positive
Bacillus	Phenol	0.45	0.224216	positive
Bacillus	Lactitol	0.433333	0.243952	positive
Bacillus	2-Hydroxycinnamic acid	0.433333	0.243952	positive
Bacillus	Benzaldehyde	0.316667	0.406397	positive
Bacillus	Phenylalanine	0.183333	0.63682	positive
Bacillus	Coumarin	0.166667	0.668231	positive
Bacillus	Threonic acid	0.166667	0.668231	positive
Bacillus	(+)-Nootkatone	0.05	0.898353	positive
Bacillus	Neotame	-0.03333	0.932157	negative
Bacillus	Phenylacetic acid	-0.05	0.898353	negative
Bacillus	Nicotine	-0.08333	0.831214	negative
Bacillus	Maltol	-0.13333	0.732368	negative
Bacillus	4-Aminobenzoic acid	-0.33333	0.380713	negative
Bacillus	Quercetin	-0.4	0.286105	negative
Bacillus	Tsugaric acid A	-0.4	0.286105	negative
Bacillus	Carylophyllene Oxide	-0.4	0.286105	negative
Bacillus	L-Malic acid	-0.41667	0.264586	negative
Bacillus	(-)-alpha-Terpineol	-0.41667	0.264586	negative
Bacillus	Aconine	-0.41667	0.264586	negative
Bacillus	8-Benzylcanadine	-0.53333	0.139227	negative
Bacillus	Isophorone	-0.56667	0.111633	negative
Bacillus	Anabasine	-0.66667	0.049867	negative
Bacillus	Cinnamaldehyde	-0.68333	0.042442	negative
Bacillus	alpha-Ionene	-0.7	0.03577	negative
Bacillus	Linalool	-0.7	0.03577	negative
Bacillus	[6]-Gingerol	-0.85	0.003705	negative
Bacillus	Neryl acetate	-0.98333	1.94E-06	negative

Table. S3 Culturable microorganisms in tobacco leaves.

**GenBank accession number**	**number**	**Strain name**	**Starch I**	**Protein I**	**Cellulose I**
OP341362	YS191	*Bacillus safensis subsp. Safensis*	-	-	-
OP341363	YS192	*Bacillus safensis subsp. Safensis*	-	-	-
OP341364	YS193	*Bacillus altitudinis*	-	++	+++
OP341365	YS194	*Bacillus altitudinis*	-	-	+

OP341366	YS195	*Bacillus altitudinis*	-	-	+
OP341367	YS196	*Niallia circulans*	-	-	-
OP341368	YS197	*Bacillus altitudinis*	-	-	+
OP341369	YS198	*Bacillus altitudinis*	-	-	+
OP341370	YS199	*Bacillus altitudinis*	-	++	++
OP341371	YS1910	*Bacillus xiamenensis*	-	-	-
OP456963	YS181	*Terribacillus halophilus*	-	-	-
OP456964	YS182	*Terribacillus halophilus*	-	-	-
OP456965	YS183	*Terribacillus goriensis*	-	-	-
OP456966	YS184	*Bacillus paralicheniformis*	-	-	++
OP456967	YS185	*Terribacillus goriensis*	-	-	-
OP456968	YS186	*Bacillus zanthoxyli*	-	-	-
OP456969	YS187	*Bacillus siamensis*	-	-	-
OP456970	YS188	*Priestia aryabhattai*	-	-	-
OP456971	YS189	*Bacillus siamensis*	-	-	-
OP456972	YS1810	*Bacillus safensis subsp. Safensis*	-	-	-
OP476334	YS171	*Bacillus velezensis*	+	-	+
OP476335	YS172	*Bacillus velezensis*	+	-	+
OP476336	YS173	*Bacillus velezensis*	+	-	+
OP476337	YS174	*Bacillus velezensis*	+	-	+
OP476338	YS175	*Bacillus velezensis*	++	-	++
OP476339	YS176	*Cytobacillus oceanisediminis*	-	-	-
OP476340	YS177	*Heyndrickxia oleronia*	-	+	+
OP476341	YS178	*Niallia circulans*	-	-	-
OP476342	YS179	*Bacillus siamensis*	-	+	+
OP476343	YS1710	*Niallia circulans*	-	-	-

OP476351	YS151	*Terribacillus goriensis*	-	-	-
OP476352	YS152	*Terribacillus goriensis*	-	-	-
OP476353	YS153	*Niallia circulans*	-	-	-
OP476354	YS154	*Bacillus tequilensis*	++	-	+++
OP476355	YS155	*Heyndrickxia oleronia*	-	+	+
OP476356	YS156	*Heyndrickxia oleronia*	-	+	+
OP476357	YS157	*Bacillus velezensis*	++	-	+++
OP476358	YS158	*Terribacillus halophilus*	-	-	-
OP476359	YS159	*Terribacillus aidingensis*	-	-	-
OP476360	YD151	*Terribacillus halophilus*	-	-	-
OP476361	YD152	*Terribacillus saccharophilus*	-	-	-
OP476362	YD153	*Terribacillus halophilus*	-	-	-
OP476363	YD154	*Terribacillus halophilus*	-	-	-
OP476364	YD155	*Terribacillus halophilus*	-	-	-
OP476365	YD156	*Terribacillus goriensis*	-	-	-
OP476366	YD157	*Terribacillus halophilus*	-	-	-
OP476367	YD158	*Terribacillus halophilus*	-	-	-
OP476368	YS141	*Terribacillus goriensis*	-	-	-
OP476369	YS142	*Bacillus amyloliquefaciens*	++	-	++
OP476370	YS143	*Terribacillus goriensis*	-	-	-
OP476371	YS144	*Terribacillus halophilus*	-	-	-
OP476372	YS145	*Terribacillus halophilus*	-	-	-
OP476373	YS146	*Terribacillus goriensis*	-	-	-
OP476374	YS147	*Heyndrickxia sporothermodurans*	-	-	-
OP476375	YS148	*Niallia nealsonii*	-	-	-
OP476376	YS149	*Heyndrickxia oleronia*	-	+	+

OP476377	YC151	*Niallia circulans*	-	-	-
OP476378	YC152	*Niallia circulans*	-	-	-
OP476379	YC153	*Heyndrickxia oleronia*	-	+	+
OP476380	YC154	*Niallia circulans*	-	-	-
OP476381	YC155	*Niallia circulans*	-	-	-
OP476382	YC156	*Niallia circulans*	-	-	-
OP476383	YC157	*Bacillus velezensis*	+	-	+
OP476384	YC158	*Bacillus tequilensis*	+	-	+
OP476385	YC159	*Bacillus velezensis*	+	-	+
OP476386	YH181	*Terribacillus halophilus*	-	-	-
OP476387	YH182	*Niallia circulans*	-	-	-
OP476388	YH183	*Terribacillus goriensis*	-	-	-
OP476389	YH184	*Niallia circulans*	-	-	-
OP476390	YH185	*Bacillus safensis subsp. safensis*	-	-	-
OP476391	YH186	*Bacillus pumilus*	+++	+++	+++
OP476392	YH187	*Bacillus cereus*	+	-	-
OP476393	YH188	*Cronobacter sakazakii*	-	-	-
OP476394	YH189	*Enterobacter hormaechei subsp. xiangfangensis*	-	-	-
OP476395	YS201	*Cronobacter malonaticus*	-	-	-
OP476396	YS202	*Enterobacter hormaechei subsp. xiangfangensis*	-	-	-
OP476397	YS203	*Klebsiella pneumoniae subsp. pneumoniae*	-	-	-
OP476398	YS204	*Enterobacter hormaechei subsp. oharae*	-	-	-
OP476399	YS205	*Enterobacter hormaechei subsp. xiangfangensis*	-	-	-

OP476400	YS206	*Enterobacter hormaechei subsp. xiangfangensis*	-	-	-
OP476401	YS207	*Enterobacter hormaechei subsp. xiangfangensis*	-	-	-
OP476402	YS208	*Enterobacter hormaechei subsp. xiangfangensis*	-	-	-
OP476403	YS209	*Pseudomonas hunanensis*	-	-	-
OP476404	YS211	*Terribacillus goriensis*	-	-	-
OP476405	YS212	*Rummeliibacillus stabekisii*	-	-	-
OP476406	YS213	*Bacillus velezensis*	+	-	+
OP476407	YS214	*Terribacillus goriensis*	-	-	-
OP476408	YS215	*Terribacillus halophilus*	-	-	-
OP476409	YS216	*Terribacillus halophilus*	-	-	-
OP476410	YS217	*Terribacillus goriensis*	-	-	-
OP476411	YS218	*Bacillus velezensis*	+	-	+
OP476412	YS219	*Terribacillus goriensis*	-	-	-
OP476413	YS2110	*Terribacillus halophilus*	-	-	-
OP476414	YS2111	*Terribacillus halophilus*	-	-	-
OP476415	YS2112	*Terribacillus halophilus*	-	-	-
OP476416	YS2113	*Terribacillus halophilus*	-	-	-
OP476417	YDB1	*Enterococcus lactis*	-	-	-
OP476418	YDB2	*Mammaliicoccus sciuri*	-	+++	-
OP476419	YDB3	*Acinetobacter variabilis*	-	-	-
OP476420	YDF1	*Bacillus velezensis*	+	-	+
OP476421	YDF2	*Bacillus velezensis*	+	-	+
OP476422	YDF3	*Bacillus velezensis*	+	-	+
OP476423	YDF4	*Bacillus velezensis*	+	-	+
OP476424	YDF5	*Bacillus wiedmannii*	-	-	-

OP476425	YDF6	*Acinetobacter baumannii*	-	-	-
OP476426	YDF7	*Bacillus licheniformis*	+	+	+
OP476427	YDF8	*Bacillus velezensis*	+	-	+

Nite: I is the degradation index, +++ indicates that the degradation index I> 4; ++ indicates that the degradation index I> 2; + indicates that the degradation index I> 1.0.

Table. S4 Comparison of the mean scores of sensory attributes and overall quality for different groups of tobacco in sensory evaluation

Sensory score / Group	aroma quality	aroma quantity	off-flavors	Concentration	strength	smoothness	irritation	dryness	aftertaste	sweetness	Weighted total score
CK	5.00 c	4.88 c	4.50 e	6.00 ab	7.00 a	4.63 e	5.00 d	5.00 e	4.50 d	4.50 f	52.95 c
T1	5.33 a	5.28 ab	5.13 b	5.88 ab	6.65 bc	5.25 c	5.50 a	5.50 a	5.00 b	5.08 b	53.99 c
T2	5.16 b	5.20 ab	4.76 d	5.96 ab	6.68 bc	4.90 d	5.10 c	5.10 d	4.80 c	4.60 e	55.71 c
T3	5.00 c	5.33 a	5.56 a	6.10 a	6.72 b	5.54 a	5.20 b	5.20 c	5.34 a	5.34 a	57.56 b
T4	5.24 ab	5.28 ab	5.00 bc	6.10 a	6.76 b	5.14 cd	5.44 a	5.40 b	5.00 b	4.80 c	54.67 c
T5	5.06 c	5.06 b	4.80 d	6.10 a	6.72 b	4.94 d	5.20 b	5.20 c	4.80 c	4.60 e	53.53 c
T6	5.06 c	5.06 b	4.80 d	5.82 b	6.54 c	4.98 d	5.24 b	5.20 c	4.84 c	4.60 e	55.74 c
C1	5.24 ab	5.24 ab	4.98 bc	6.00 ab	6.62 bc	5.08 d	5.24 b	5.20 c	4.98 b	4.74 d	56.19 c
C2	5.2 ab	5.24 ab	4.90 c	6.10 a	6.68 bc	4.94 d	5.20 b	5.20 c	4.84 c	4.60 e	57.45 b
C3	5.4 a	5.40 a	5.14 b	5.90 ab	6.4 c	5.4 b	5.60 a	5.60 a	5.20 a	4.90 c	58.96 a
C4	5.3 a	5.30 a	5.10 b	5.80 b	6.38 c	5.43 b	5.63 a	5.63 a	5.00 b	4.83 c	54.38 c
F	5.38 a	5.38 a	5.13 b	6.05 a	6.65 bc	5.25 c	5.63 a	5.63 a	5.13 a	5.25 a	59.75 a

Note: Unique lowercase letters within the same column indicate significant differences between different tobacco varieties, P< 0.05.

Figure. S1

PCA and OPLS-DA scores of positive and negative ion patterns in different fermentation years (increasing in turn). (A) and (B) One-year positive and negative ion pattern volcanic map of fermentation; (C) and (D) Two-year positive and negative ion pattern volcanic map of fermentation;(E) and (F) Three-year positive and negative

ion pattern volcanic map of fermentation; (G) and (H) Four-year positive and negative ion pattern volcanic map of fermentation;A1-A4, FCT aged 0-4 years.

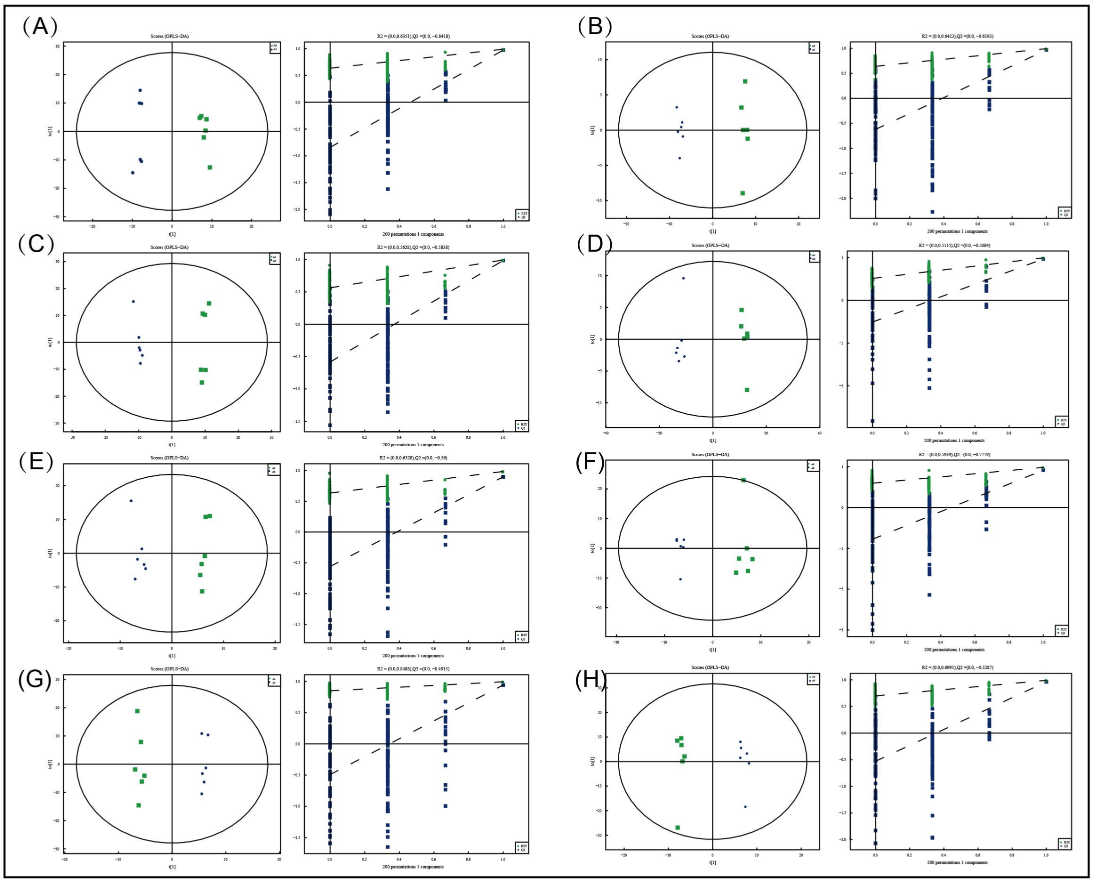

Figure. S2 Volcanic diagram of tobacco metabolites expression is derived from the UHPLC-Q-TOF MS metabolite spectrum of tobacco. (A) and (B) One-year positive and negative ion pattern volcanic map of fermentation; (C) and (D) Two-year positive and negative ion pattern volcanic map of fermentation;(E) and (F) Three-year positive and negative ion pattern volcanic map of fermentation; (G) and (H) Four-year positive and negative ion pattern volcanic map of fermentation; A1-A4, FCT aged 0-4 years.

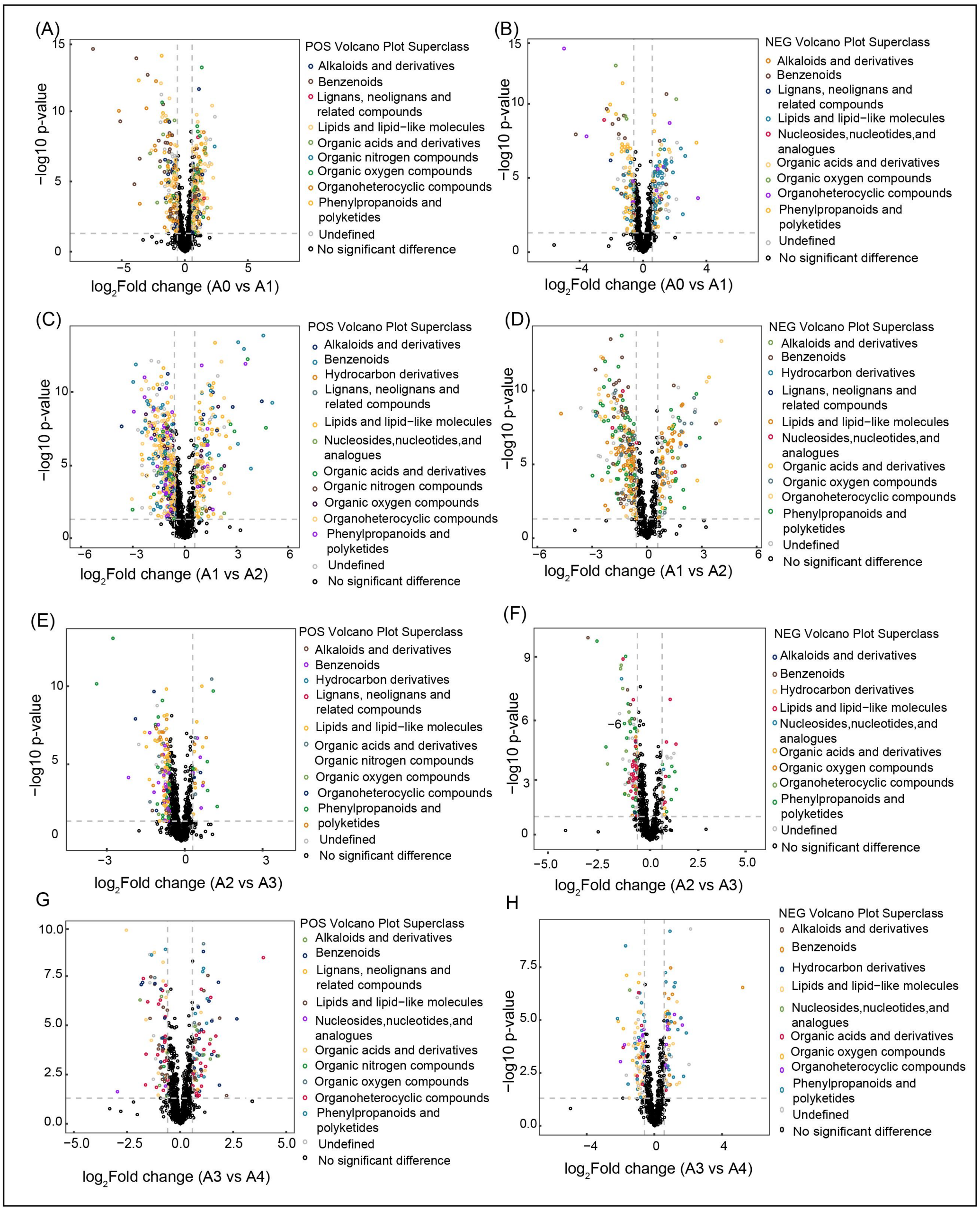

Figure.S3 Hierarchical clustering analysis and heat map of the differential metabolites of flue-cured tobacco identified under positive ion. A1-A4, FCT aged 0-4 years.

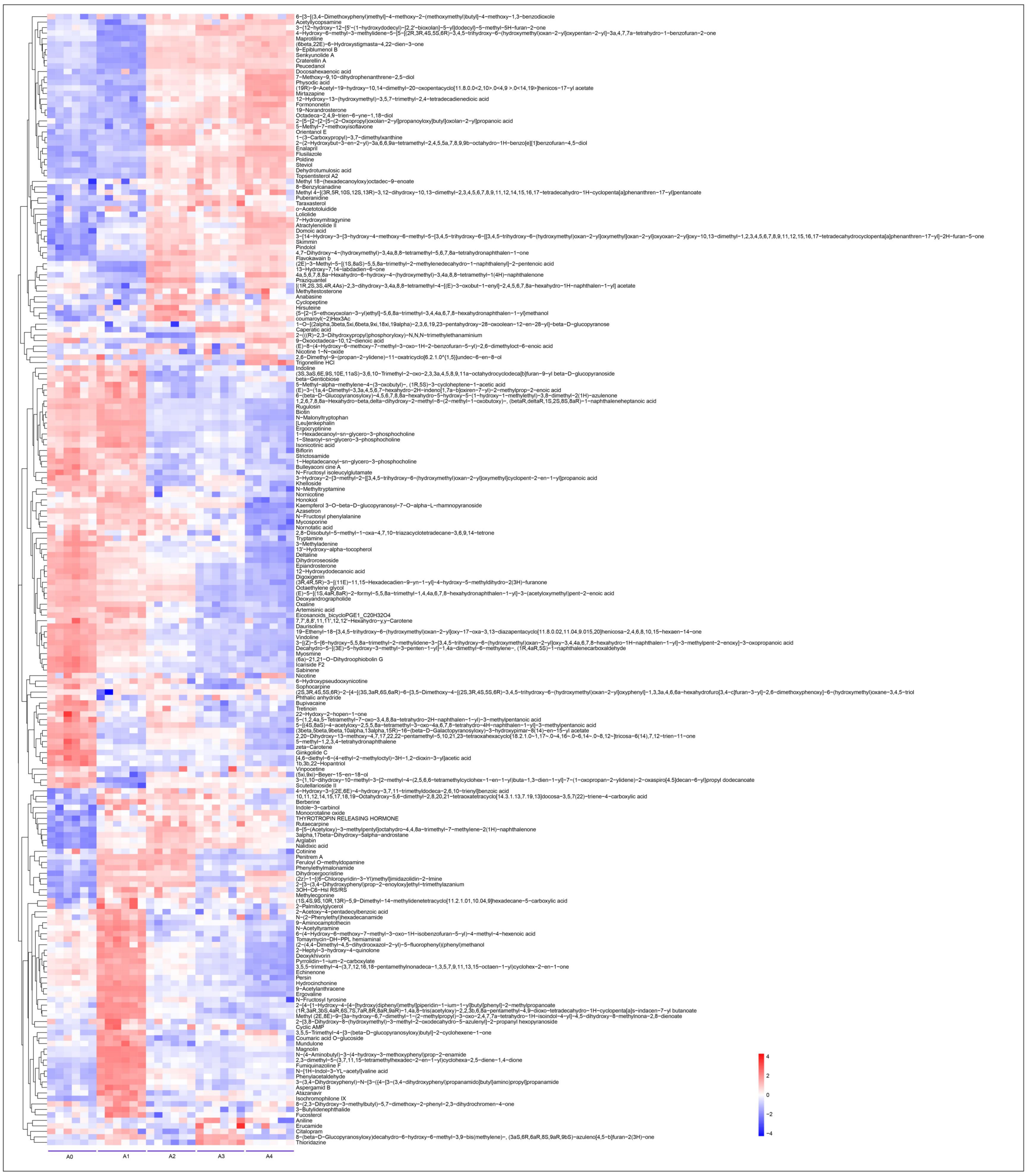

Figure. S4 Hierarchical clustering analysis and heat map of the differential metabolites of flue-cured tobacco identified under negative ion mode. A1-A4, FCT aged 0-4 years.

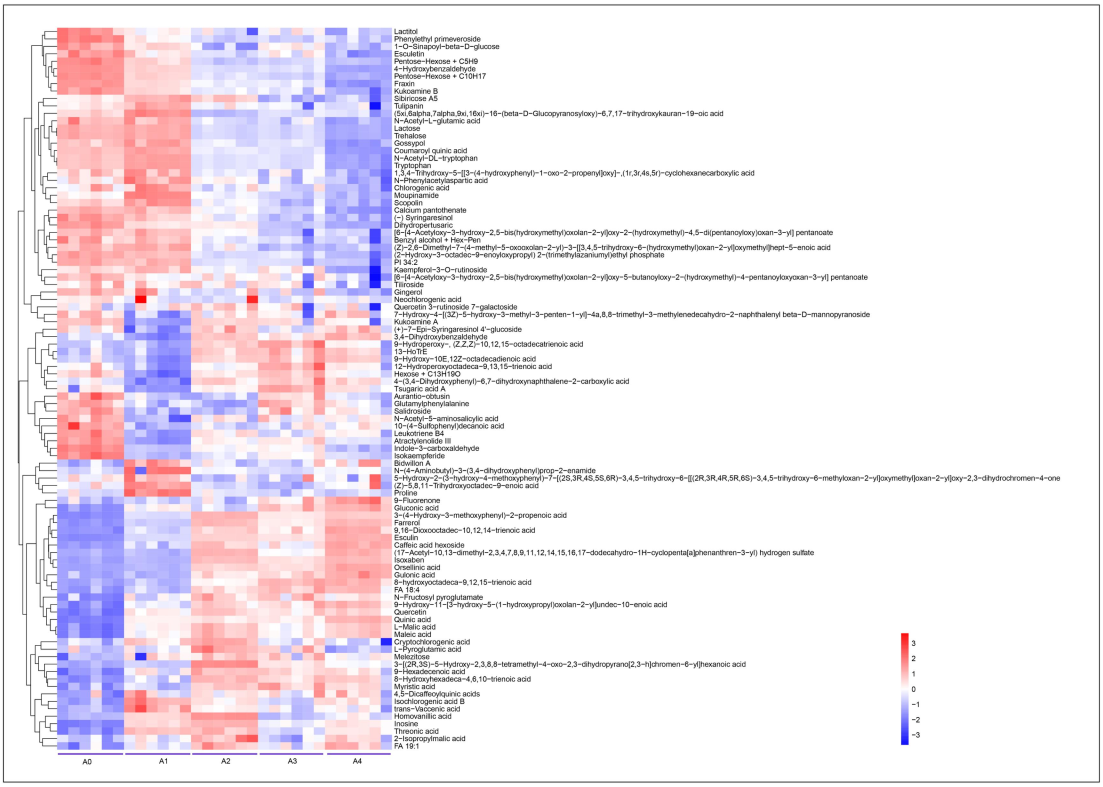

Figure. S5 PCoA analysis of tobacco leaves during fermentation. Points of the same color represent the parallel groups of the samples, and the distribution status of the points reflects the divergence degree of the samples. A1-A4, FCT aged 0-4 years.

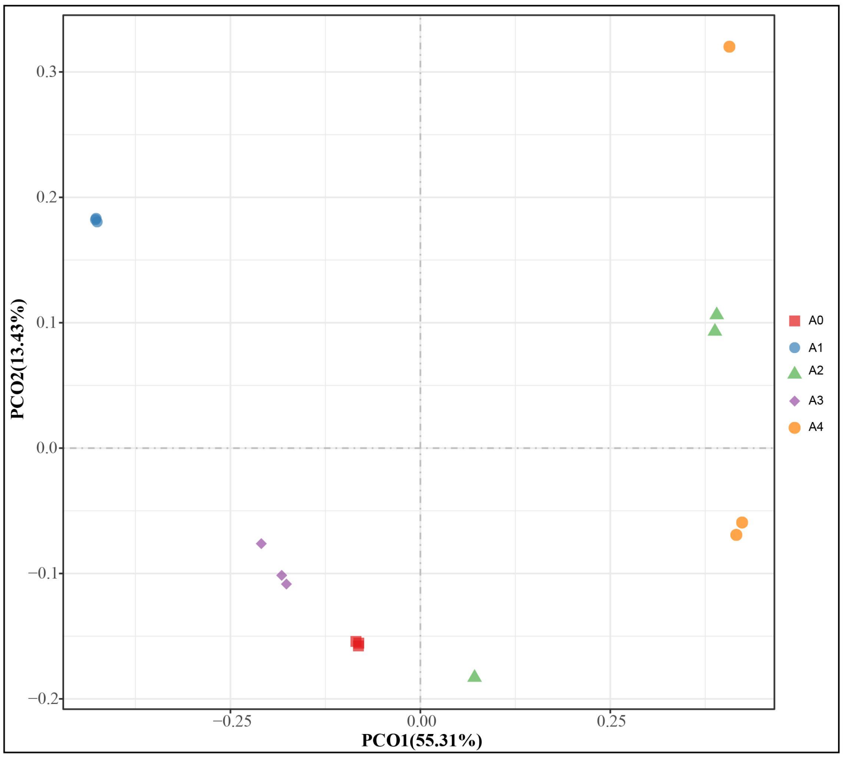

Figure. S6 Spearman correlation analysis between the relative abundance of the top 20 microbial genera in FCT fermentation samples and metabolic substances across different groups. A1-A4, FCTs aged 0--4 years.

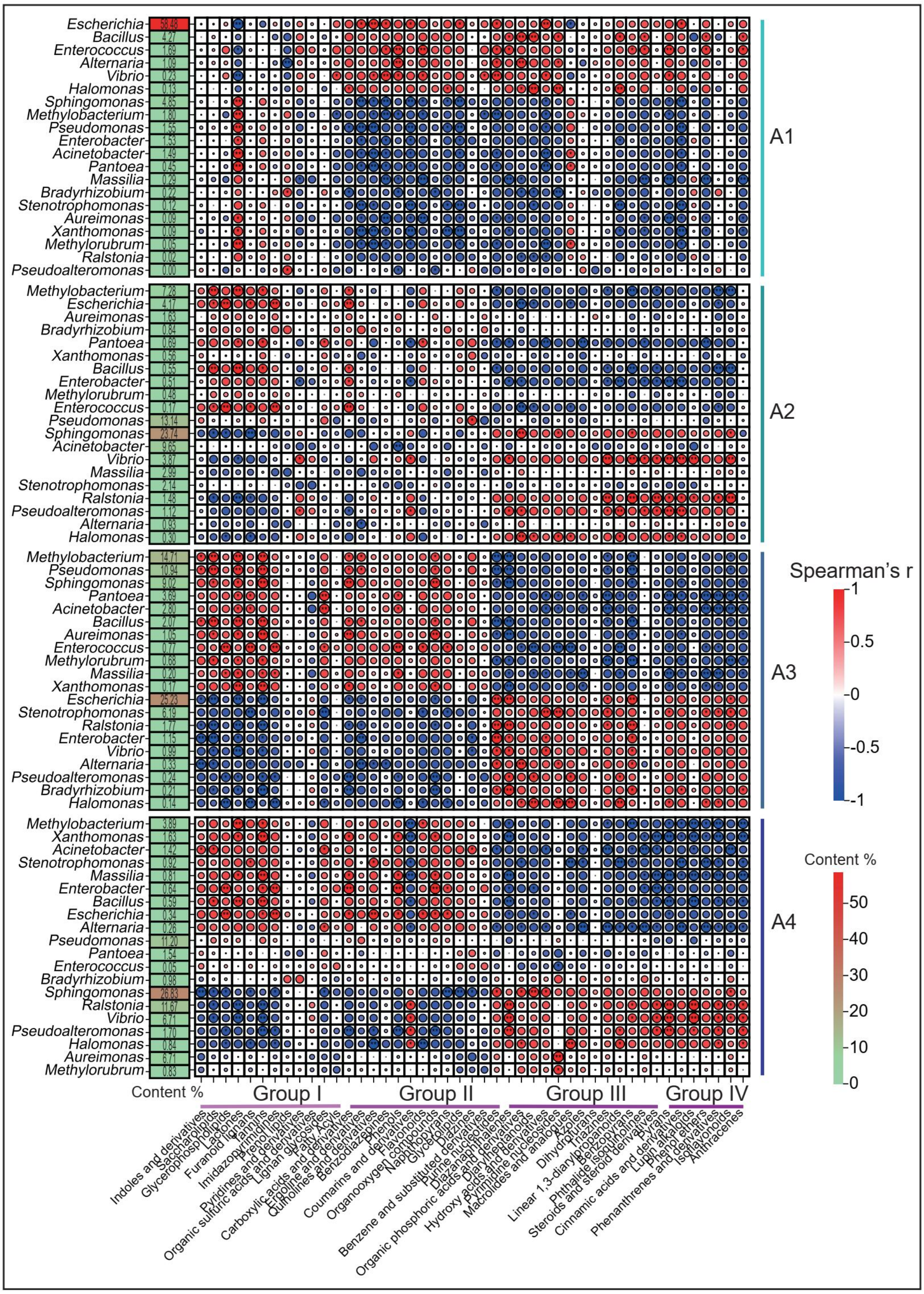

Figure. S7 Schematic diagram of some fragrant components and arom atic amino acids metabolism

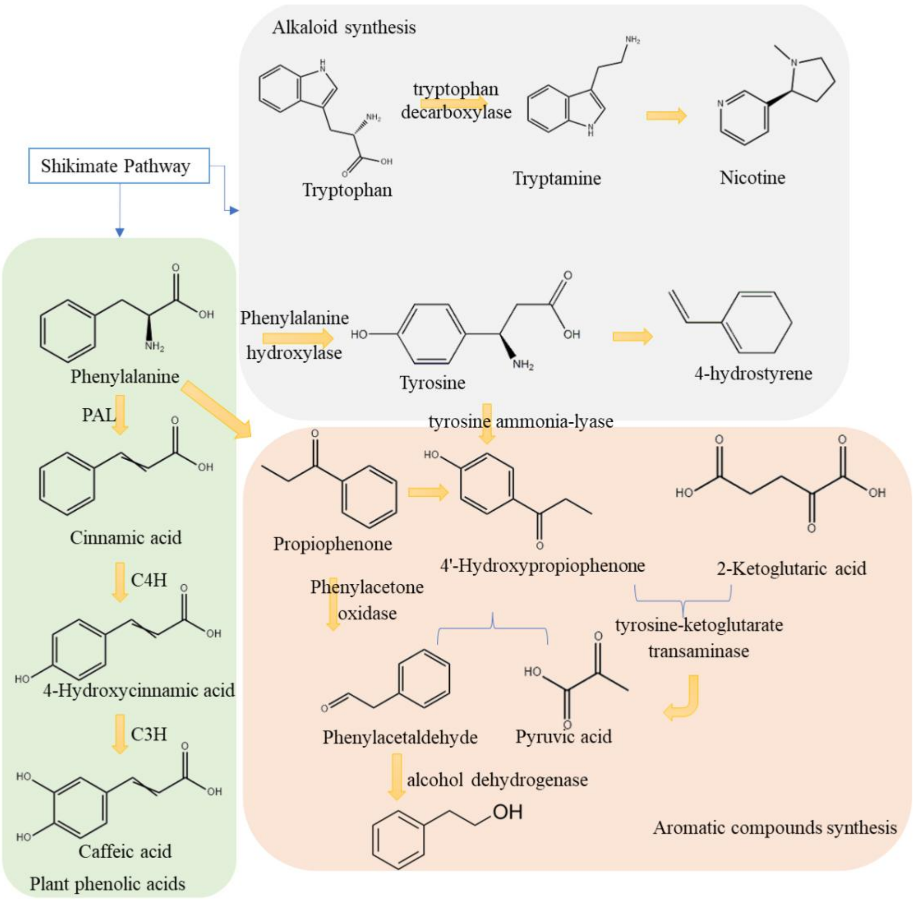

Supplement: Supplementary file 2 [file DataSheet1.pdf]
